# Supplementary material for: Magnitude and factors associated with appropriate complementary feeding practice among mothers of children 6–23 months age in Shashemene town, Oromia- Ethiopia: Community based cross sectional study
Source: PLoS One. 2022 Mar 29;17(3):e0265716. doi: 10.1371/journal.pone.0265716 (PMC8963544; doi:10.1371/journal.pone.0265716)
Supplement: S1 File — (ZIP) [file pone.0265716.s001.zip › supporting information/afaaan oromoo version of document completered.docx]

## Subject Information Sheet (Afaan Oromo Version)

Akkam jirta? Maqaankoo______________________________jeedhama.Ammaqu’anoo University MWU , Dipartimantii Fayyaa Hawaasaa keessatti adeemsifamu fi fedhi keeyoo ta’ee gaafii kanaaf ofii keessani deebistu .Sababiin qu’annoo kanaa s haal raawii nyaata dabalata fi sadarka nyaata da’ima umuri jia’a 6 hanga jia’a 23 godina arsii lixati qorannoo hawassa keesati gageefamu dha. Kanaafuu dhima armaan olitii ibsame irrati gaafillee tokkoo tokkoon isa akka deebiftan barbaada. Buu’an qu’anoo kunis dhimma armaan olitti ibsame iratti namoota seera fi karroora baasanii fi gargaarssa guddaa akkata’u ni hubachiisna. Hirmaanaan keetillee as keessatti bu’aa guddaa akka ta’u ni’abdana. Hirmaanaan keetien walqabatuu ammo sodaan wayiituu akka sitti hin uumamnee ni beeksisna. Maalif yoo jeette Gaafilee irratti maqaan kee hin barreefamu waan ta’eef, kanaafuu icciitiin sirriitti eegama.Akasumas ammo feedhii keemalee hirmaatuuf dirqama hin qabdu.Gaafileen kun hanga sa’aatii daqiiqaa___________ fudhachuu ni danda’a.

##

## 8.4. Oral Consent Form ( Afaan Oromo Version)

Waraqaa oddefannoo qu’annoo kana ilaalchisee armaan olitii kan barraaye dubbiseenaa dubbisamees hubannoo keessa galchee jiraa, wanta narraa barbaadamus naaf galleejiraa. Ida’amaanis wayitiin barbaadameeti sababii tokkoo mallee osooana fi maati kiyaratti rakko hin uumin addan kutuu akkan danda’uullee beekee jiraa.

Mallattoo hirmaataa ________________ guyyaa ____________________ Mallattoo abaa gaafii dhiiyeesee mirkaneesu .Maqaa _________________________ Mallattoo _______________ guyyaa____________

Amma gaafii itti fufuu danda’a?

Eyeeni_____________ittifufuu

Lakki______________gaafachudhiisuu fi gaafiif deebisaa galateefachuu.

##

**Maqaaa _**___________________ ___________**Telephone** _________

| Gaafile Afaan oromoo | | | | | Kodi------------ | |  |  |
| --- | --- | --- | --- | --- | --- | --- | --- | --- |
|  | | | | | Magaalaa 1 | |  |  |
| Yooni Umriin daima guyyaa 183-730 ta’a gaafi ittifufi | | | | | Ganda ______ | |  |  |
| Guyyaa / Ji’an / Waggaa (A.L.H):------/----/----Odefannoo itti funaname | | | | | | |  |  |
| 1. Umrii haadhaa _____________ | | |  | | | |  |  |
| 1. Sadarkaa barnootaa haadhaa | | |  | | | |  |  |
|  | | | 1. Barnoota bu’uraa 2. Sad. 1ffaa 3. Sad. 2ffaa 4. Barnoota Olaanaa | | | |  |  |
| 1. Saali da’imaa maali? | | | 1. Dhiira 2. Dhala | | | |  |  |
| 1. Umriin da’ima Ji’an meeqa? ibsi | | |  | | | |  |  |
| 1. Sabani kee maali? | | | 1. Oromoo 2. Amaaraa  3. walaayitaa 4. Tigree 9. Kanbira ibsi-- | | | |  |  |
| 1. Gosa hojjii haadha mana | | | 1, Haadha warra 2. hojatuu manaa  3.hojatu mootumaa 4. Barataa/tu  5. daldaltu 9. Kanbiroo ibsii | | | |  |  |
| 1. Amantii kessan maali ? | | | 1. Muslimaa 2. Orthodoksi 3. kaatolik  4. prootestaantii 9. Kanbiroo ibsii | | | |  |  |
| 1. umrii amma itti deesse meqaa?------- | | |  | | | |  |  |
| 1. Sadarkaa barnootaa Abaa manaa meeqa? | | | 1. Barnoota bu’uraa 4. Sad. 1ffaa 2. Sad. 2ffaa    3.Barnoota olaanaa | | | |  |  |
| 1. Haali ga’aila hadhamana maali? | | | 1. Tanherumte 2. Tanhinherumin  3. Tangargarbate 4. Tan irradu’ee 5. Iddo adda adda jiratan | | | |  |  |
| 1. Gosa hojjii Abbaa mana maali? | | | 1.hojataa mootumaa 2. Qotee bula 3.daldala 9. Kanbiroo ibsii | | | |  |  |
| 1. Mana eenyutu bulcha/dursa? | | | 1. Dhira 2. Dhala | | | |  |  |
| 1. Galiin maati kessanii jia’an meqa? Qr ibsi | | |  | | | |  |  |
| 1. Nyaata akkam filatu? ---- | | | 1. Horii irra kan ta’e 2. Kan midhanirra ta’e | | | |  |  |
| 1. Kanaan dura ulfoftee beekta? Inni deesse kan lubbu hinqabne yoo ta’ele. | | | 1. eeye 2. hinbeku | | | |  |  |
| 1. Ulfa meeqaffati Kuni? ---- | | |  | | | |  |  |
| 1. Daa’imti dhumarra dhalatte(ji’a 6-23) dhala meeqaffaadha? | | | 1.1 ^ffa^ 2.2 ^ffa^ 3.3 ^ffa^  4 . 4 ^ffa^ 5. 5 fi ^isa ol^ | | | |  |  |
| 1. Tajajila qoranno ulfa ‘ANC’ hordaftee beekta?- | | | 1 .eeyee 2. Hinhordafne | | | |  |  |
| 1. Siaa meeqa tajaajila qoranno ulfa ANC fayyadamte? | | |  | | | |  |  |
| 1. Yoom tajaajila ANC jalqabde? | | | 1. Ji’aa1-3 2.jiaa 4-5 3. 6fi isa’ol | | | |  |  |
| 1. Ijoolee meeqa deese ? | | | ----- | | | |  |  |
| 1. Da’aima eessatti deesse ? | | | 1. manajirenyaa 2. Manayaala | | | |  |  |
| 1. Eenytu sideessisee? | | | 1. ogessa fayya 2. Ogetti gandaa 3. Kanbiro ibsi? | | | |  |  |
| 1. Karooran keetin deesse? | | | 1. eeye 2 .Miti | | | |  |  |
| 1. Akkamit deesse? | | | 1. Cininsudhaan 2. Baqaqsaan (operationiidhan) 3.kanbiroo | | | |  |  |
| 1. Yeroo meeqa adaan turtee desse ? ---- | | |  | | | |  |  |
| 1. Tajajila da’umsa booda hordoftee beekta? | | | 1. eeyee 2. Hinbeku | | | |  |  |
| 1. (A)Yoon hordofte jirate yeroo kam guyyaan ibsi? | | | 1.0-2 2. 3-6 3.7 fi isa ol | | | |  |  |
| 28(B) Harma hoosiftee beekta daa’ima? | | | 1. eeye 2. hinbeku | | | |  |  |
| 29. Erga deesse booda harma yoom daa’ima kennite? | | | 1. akkuma dhalateti  2. Dhalate Sa’aati tokko kessati  3. Dhalate sa’aati 24 kessati  9. Kanbiro (ibsi)----- | | | |  |  |
| 1. Aanan Harma hamma yoomi (hanga wagga meqa’atti) daa’ima kee hoosifte? ji’an ibsi? | | |  | | | |  |  |
| 1. Harma akka feedhi da’iamaatti yeroo yeroon dabalaa hang wagga lamaati ykn isa olti kaniteetaafi? | | | 1. eeyee 2. hinkenineefi | | | |  |  |
| **Nyaata dabalata kenuu ilaachisee (Introduction of solid, semisolid or soft foods)** | | | | | | |  |  |
| 1. Da’imti kaleesa galgala ykn guyya nyaata dhangala’a,nyaata bula’a(marqaa),nyaata jajabaa fkn-Budeena) nyaate beeka/ti ? 1. eeyee 2. Hinbeku | | | | | | | 1. **eeyee 2. hinbeku** | |
| 1. Yooni nyaate ta’e maali nyaate (adaa baasi ibsi)? | | | | |  | |  |  |
| 1. Daa’imti yeroo meeqa guyaati nyaata jajaba,lalaafa,   gidugalessa nyaate? Dhangala’a kan hin ta’in. | | | | |  | |  |  |
| 1. Daa’imafi kaleesa galgala ykn guyyaa xuuxoon nyaata(mooqa,aanan …) keniteetaafi? | | | | | 1. eeyee 2. Hinbeku | |  |  |
| 1. Daa’mti dhibee baassan qabamee beeka? | | | | | 1. eeyee 2.hinqabamne | |  |  |
| 1. Daa’imti dhibee “ART”fkn qufaa,qorra ) qabamee beeka? | | | | | 1. eeyee 2.hinqabamne | |  |  |
| 1. Daa’imti dhibee hoo’an qabamee beeka? | | | | | 1. eeyee 2.hinqabamne | |  |  |
| 1. Yoo daa’ima dhukbsate nyaata dabalataa maalgootu? | | | | | 1. Yeroo itti dedebi’e nyaatu fi qabiyee ni xiqessina 2. akkuma yeroo hundatti kennamaafi 3. itti daballe kaninaafi 4. hin latamuuf | |  |  |
| 1. Akka aada fi baratama naanoti nyaatni daa’imaaf hinkenamne jira? | | | | | 1.eeyee 2. Miti | |  |  |
| 1. yoo jira jette maali? | | | | |  | |  |  |
| 1. Akamiti Nyaata dabalata qopheesitu ? | | | | | 1.daa’imaa qobaati  2. maati waliin | |  |  |
| 1. Nyaata dabalata kami daa’imaaf filatu? | | | | | 1.kan manati qopha’u  2.kan gabaara bitamu | |  |  |
| 1. Nyaata dabalata isa kam daa’imaaf filatu | | | | | 1.daaboo  2.kuduraafi fuduraa  3.Aanan  4.juusii | |  |  |
| **Nyaata gosa adda adda nyaachisuu ilaalchisee (Minimum dietary diversity)** | | | | | | |  |  |
| 1. Da’imti kaleessa guyyaa ykn galgala (sa’ati 24 keesati) nyaata ‘grain,root & tubers’ kan akka marqaa,ruuzi,daaboo,nyaata biro kan midhaanirra hojatame nyaatera? 1.eeyee 2.Hinnyaane | | | | | | | 1. **eeyee 2. Hinqabamne 3.hinyadadhu** |  |
| 1. Daa’imti kaleessa guyyaa ykn galgala sa’ati 24 keesati nyaata baaqela fi atara ,shumburaa ,lawuzii (ocholooni) irra qopha’e( legumes & nut )nyaate beekaa ? | | | | | | 1. eeyee 2. hinnyaanne |  |  |
| 1. Daa’imti kaleessa guyyaa ykn galgala sa’ati 24 keesati (Aanani ,itituu, ayibee) dhugee beekaa ? | | | | | | 1. eeyee 2. hinbeeku |  |  |
| 1. Daa’imti kaleessa guyyaa ykn galgala sa’ati 24 keesati nyaata fooni(kan akka fooni lukuu,ra’ee horii,hoolaa, dakiyye nyaate beekaa ? | | | | | | 1. eeyee 2. hinbeeku |  |  |
| 1. Daa’imti kalessa guyyaa ykn galgala sa’ati 24 keesati nyaata foon qurxumii, nyaate beekaa ? | | | | | | 1. eeyee 2. hinbeeku |  |  |
| 1. Daa’imti kalessa guyyaa ykn galgala sa’ati 24 keesati nyaata kilee nyaate beeka? | | | | | | 1. eeyee 2. hinbeeku |  |  |
| 1. Daa’imti nyaata dubbaa(dabaaqula),karooti,sukar dinchi(maxaaxishii) nyaate beeka ? | | | | | | 1. eeyee 2. hinbeeku |  |  |
| 1. Dinichaa,boyyina ,kan biro ‘root’ irra argame nyaate beeka? | | | | | | 1. eeyee 2. hinbeeku |  |  |
| 1. Daa’imti Kuduraa magariisa(shaana,qoosxaa,salaxaa) (dark green leafy vegetables) nyaate beeka? | | | | | | 1. eeyee 2**.** hinbeeku |  |  |
| 1. Daa’imti kudura biro nyaate beeka? | | | | | | 1. eeyee  **2.** hinbeeku |  |  |
| 1. Daa’imti nyaata foon qaamaa horii/hoola/re’ee (organ meat) kan akka kale,tiruu,onnee,kan biro)nyaate beeka? | | | | | | 1. eeyee 2. hinbeeku |  |  |
| 1. Daa’imti nyaata zeeyta,dhadhaa qabu irraa hojatame nyaate beeka? | | | | | | 1. eeyee 2. hinbeeku |  |  |
| 1. Daa’imti nyaata mia’awa kan akka karameelaa buskuta ,keekii ’’chocolates, sweets, candies, pastries, cakes, or biscuits ‘’ adda addaa nyaate beeka? | | | | | | 1. eeyee 2. hinbeeku |  |  |
| 1. Nyaata kan akka maangoo ,papaya bilchaata ,qaara,timaatima ‘vitamin A ‘ badhaadhe’ (Ripe mangoes, ripe papayas)nyaate beeka? | | | | | | 1. Eeyee 2. Hinbeku |  |  |
| 1. Daa’imti nyaata firaafire biroo fi (‘fruits and vegetables ‘) guyya qoranno duratti nyaate beka? | | | | | | 1. Eeyee 2. Hinbeku |  |  |
| 1. Qabeenya kessan irratti entu murtesa? | | | | | | 1. Abba mana 2. Haadhamana 3. waliin |  |  |
| 1. Odeffanno waa’ee fayida harmahosisuu eessa argate?) | | | | | | 1. TV 2. Radio 3. Dubisuun  4. Ogessa fayya. 9. Kanbiro( ibsi)? |  |  |
| 1. Maddi odefannoo nyaata dabalata magaala irratti gurguramu essa argatte? | | | | | | 1. Ogessa fayyairra 2. Maatii   3.Midaa 4.firarraa |  |  |
| 1. Bekumsa waa’ee albuuda iyranii(iron) nyaata dhiga kennu iirati qabdu | | | | | | 1. Eeyee 2. hinbeku |  |  |
| 1. Fayidaa waa’ee Ashaboo iodinii qabuu beektu? | | | | | | 1. Eeyee 2. hinbeku |  |  |
| **Guyyaati yeroo meeqa dedebi’ani nyaata daa’imaf kenu? (Minimum meal frequency (MMF))** | | | | | | |  |  |
| 1. Daa’ma harmaa hodhufi si’a (yeroo) meqaa nyaata dabalataa keleesa kennitaniifi ? | | | | | |  |  |  |
| 1. Da’imti kalessa guyya ykn galgala anaan harmaa kan nama biraa dhugude beekti (kubaayaan/faldha’nan/? | | | | | | 1. Eeye 2. hinbektu |  |  |
| 1. Kalessa daa’ima harma hinhoneefi sia (yeroo) meqaa nyaata daa’imaa kennitan? | | | | | |  |  |  |
| 1. Kalessa da’aimti harmahoodhu umriin ji’a 6–23 nyaata Marqaa, firfirii budeena, ykn nyaata lalaafa biro kennamefi jira?’ /receive solid, semi-solid, or soft foods during the previous day?’ | | | | | | 1. Eeye 2. hinbektu |  |  |
| 1. Kaleessa da’aimti harma hinhoone kan umriin ji’a 6–23 nyaata jajabaa, jidugala ykn lalaafa aanan dabalate kennamefi jira?’ | | | | | | 1. Eeyee 2. hinkanamneef |  |  |
| 1. Daa’imaaf yoomi /Ji’a meeqa irratti nyaata dabalataa kenitaniif ? | | | | | |  |  |  |
| 1. Yoo nyaata ji’a jahaan dura jalqabdaniif maaliif ? | | | | | |  |  |  |
| 1. Ulfini daa’ima amma meqa? Madaali? | | | | | |  |  |  |
| 1. Dheerini daa’ima amma hagam ,Madaali? | | | | | |  |  |  |
| 1. Maddi bishaanii dhugaatii baayyinaan kan miseensota abbaa warraa kanaa maali? 2. ujummoo/Boono 3. lafa keessa ujummoodhaan dhufu 4. harkaan qotamee bahame 5. lafa keessaa burqitu/burqituu 6. roobaa/bokkaa kuusuudhaan 7. lafarraa yaa’an (laga, hidhaa, burqaa ykn   jalisiiti  fayyadamuu) 8. warshaatti oomishame 9. Kan biroo(ibsi)------- | | | | | | |  |  |
| 1. Manajirenya kessan kan Maali? | 1. Kan dhunfa 2. kan kira | | | | | |  |  |
| 1. Mana fincaanii isaani kami?   (Ilaali galmeesi) | 1. Fooyya’aa qilleensa xiraa’aa baasu (VIP) 2. ejjannoo miilaa qabu 3. ejjannoo miilaa hin qabne 4. koompoostiif oolu 5. Baaldii/Meeshaa itti hagamu ykn fincaanamu 6. rarra’aa/Hanging toilet/ 7. hin jiru/dirretti ykn bakketti fayyadamu 8. Kanbiro( ibsi) | | | | | |  |  |
| **Meeshaalee Ijoo manni irraa ijaarame Ilaallii galmeessi.** | | | | | | |  |  |
| 1. Lafa/floor/ manaa uumamaa | | 1. Biyyee       3.   Dhoqqee 2. Xaawulaa 4. Leemman/qarkaa | | | | |  |  |
| 1. Bu’ura lafaa mana keesa jiraatanii | | 1. muka bocame  (Xaawula)   2.   Shakilaa(ceramic tiles )   1. Simintoo 2. Kan biroo (Ibsi) | | | | |  |  |
| 1. Wantoota ijoo baaxiin manaa irraa hojjatame | | 1. qorqooro     3.  Citaa 2. Biyoon baaxii irra kaa’ame | | | | |  |  |
| 1. Wantoota ijoo dhaabni manaa irraa hojjatame. | | 1. sibiila 2. Muka /Leemman/Qarkaaha 3. Kan biro ibsi | | | | |  |  |
| 1. Bu’uraa Dhaaba manaa | | 1. Shambaqqoo dhoqqeen 2. Dhagaa dhoqqeen 3. Bilookkeettii dhoqqee irraa hojjatame garuu hin uwwisamne 4. Komborsaatoo/cardboard,plywood/ 5. Muka deebi’amee itti fayyamame/reused/ | | | | |  |  |
| 1. Dhaaba mana dhumaa/Finished wall/ | | 1. Simintoo 2. Dhaga simintoon 3. Xuubii/Bricks/ 4. Bilookettii dhoqqee irraa hojjatamee uffifame/Covered adobe/ 5. Muka dhaabaa tolchame/wood planks/9. Kan biro Ibsi | | | | |  |  |
| 1. Mana jirenyaa ilaachise Baayinna kutaalee meeqa qabdan? | | | | 1. Kutaa tokko qofa 2. Kutaalee lama 3. Kutaa lamaa ol | | |  |  |
| 1. Kutaa meeqa ciisichaaf itti fayyadamtu? ------------ | | | |  | | |  |  |
| Maatiin keessan ykn namni asi keessa jiraatu wantoota asii gaditti tuqaman  kamiin qabu:   1. Eleektiriikii 2. Raadiyoonii 3. Teeleeviizhiinii 4. Bilbila Moobaayiliin Alaa 5. Firiijii 6. Miinjaala 7. Teessoo 8. Siree 9. Eelee Elekitiriikaa 10. Keeroosiinii YknDhiibbaa 11. Laampii 12. Sa’aatii 13. Moobaayila   Yoo 1 gad ta’e “00”galmeessi. yoo 95fi isa ol ta’e ‘95’galmeessi. yoo hin beekkamne ‘99’ galmeessi. | | | | 1=eeyyee              2=hinqabu  1=eeyyee              2=hinqabu  1=eeyyee              2=hinqabu  1=eeyyee              2=hinqabu  1=eeyyee              2=hinqabu  1=eeyyee              2=hinqabu  1=eeyyee              2=hinqabu  1=eeyyee              2=hinqabu  1=eeyyee              2=hinqabu  1=eeyyee              2=hinqabu  1=eeyyee              2=hinqabu  1=eeyyee              2=hinqabu  1=eeyyee              2=hinqabu | | |  |  |

**Waa’ee argatinsa nyaataa ilaala(Household Food Insecurity Access Scale (HFIAS) )**

Gaafi hunda namooni yeroo torbe afur ykn guyyaa 30 kessattii nigaafatamu? Deebi eeyee ykn Miti ta’a. yooni deebin eeyee ta’e, gara gaafi yeroo meeqa akka ta’ee gafadhu: 1= **rarely** (yeroo tokko ykn lama turban 4n darbe kessatti), 2= **Sometimes**/ yeroo tokko tokko(yeroo sadi hama kudhani),3=**often**/ yeroo hedu(yeroo Kudhani oli turban 4n darbe kessatti) yeroo torbee 4n darbe kessatti.(In the past 4 weeks,) fkn, Maatin kessan nyaata ga’a dhabuun yaadawee beeka?

| **Lkk.** | **gaafile taa’te illachisee ( Occurrence Questions)** | | Debin miti taanan  gaafi itti aanuti darbi | | Yeroo meeqa/ How often? |
| --- | --- | --- | --- | --- | --- |
|  | Torbee afran dabre kessatti , Maatin keessan nyaata ga’a dhabuun yaadawee beeka? | | Eeyye              1  Miti              0 | | 1. yeroo tokko/lama 2. yeroo tokko tokko 3. yeroo hedu |
|  | Sababaa irrina qabeenyatin, torbe afran darbe kessatti nyaata isin filatan maatii kessan kessaa kan hin nyaatin /fayadamuu dadhabee jiru /a? | | Eeyye              1  Miti              0 | | 1. yeroo tokko/lama 2. yeroo tokko tokko 3. yeroo hedu |
| 3 | Torbe afran darbe kessatti sababaa irrina qabeenyatin gosa addaddaa kan hinqabnee/gahaa kan hintaanee nyaatani jiru? | | Eeyye              1 Miti              0 | | 1. yeroo tokko/lama 2. yeroo tokko tokko 3. yeroo hedu |
|  | sababaa irrina qabeenyatin kan ka’e ,torbe afran darbe kessatti maatii kessan keessa nyaata otto hin jalatin kan nyaatan jiru? isiins dabalatee | | Eeyye              1  Miti              0 | | 1. yeroo tokko/lama 2. yeroo tokko tokko 3. yeroo hedu |
|  | sababaa nyaatat ga’a dhabutin ,torbe 4n darbe kessatti maatii kessan kessa nyaata xinno/a kan hanga nyaachuu barbaadni gadii nyaatan jiru? | | Eeyye              1  Miti              0 | | 1. yeroo tokko/lama 2. yeroo tokko tokko 3. yeroo hedu |
|  | sababaa nyaatat ga’a dhabutin ,torbe 4n darbe kessatti maatii kessan keessa guyatti yeroo itti nyaachuun baratamee gadi nyaatame beeka? | | Eeyye              1  Miti              0 | | 1. yeroo tokko/lama 2. yeroo tokko tokko 3. yeroo hedu |
|  | Torbe 4n darbe kessatti nyaani nyaatamu bifa kamu ta’e dhabame beeka sababaa qabeenya ga’a dhabutin? | | Eeyye              1 Miti              0 | | 1. yeroo tokko/lama 2. yeroo tokko tokko 3. yeroo hedu |
|  | Torbe 4n darbe kessatti nyaata otto hin nyaatin beelan/shoome kan bule sababaa nyaata ga’a dhabutin jira? | | Eeyye              1  Miti              0 | | 1. yeroo tokko/lama 2. yeroo tokko tokko 3. yeroo hedu |
|  | Torbe afran darbe kessatti humaa/nyaata otto hin nyaatin beelan/shoome kan guyyaa fi halkan guutu ole bule sababaa nyaata ga’a dhabutin jira? | | Eeyye              1  Miti              0 | | 1. yeroo tokko/lama 2. yeroo tokko tokko 3. yeroo hedu tokko |
| Kanati aansuun kaleessa nyaata dhugamu, guyyaa ykn galgala daa’imaaf kename isin gaafadha?  Nyaata armaan gaditi tareefame keesa kamtu laatameef?  Next I would like to ask you about some liquids that ***(NAME)*** may have had yesterday during the day or at night. | | | | | |
|  | Bifa nyaata | CODING CATEGORIES | | Frequency consuming (yeroo meeqa) | |
|  | Daa’imti bishan dhuge beekti | 1. eeye 2. hindhugne | |  | |
|  | Nyaata xaasaan qophaa’ee kennitaniifi beektu? Infant formula such as [insert local examples]? | 1. eeye 2. hindhugne | |  | |
|  | Aanan : -hori, xasaa,harcee loonii hoa’a dhugde? | 1. eeye 2. hindhugne | |  | |
|  | Jiwsii dhudee beekti? | 1. eeye 2. hindhugne | |  | |
|  | Shorbaa dhugee beekti? | 1. eeye 2. hindhugne | |  | |
|  | Itittu dhugdee beekti? | 1. eeye 2. hindhugne | |  | |
|  | Axmitii ykn Mooqa qallaa dhugdee beekti? (Thin porridge) | 1. eeye 2. hindhugne | |  | |
|  | Kan biroo kan obaastan yoo jirate ibsi? |  | |  | |

1. Eeyee 0. Miti , deebin miti yoota’e, ( No) (skip to Q2 ykn gaafi lamatti darbi)

Deebin 1 = Yes /eeyee yota’ee yeroo meqaaf akka ta’ee gafadhu?/

|  | Sadarka barnoota haadha | | Umurii haadha |
| --- | --- | --- | --- |
|  | 1ffa | 2ffaa |  |
| Daa’imiti yoo nyaata nyaatu grgaarun barbaachisaadha? |  |  |  |
| Daa’ima suuta jedhee obsaan nyaachisuun barbaachisaadha |  |  |  |
| Daa’ima kiyaa nyaata akka nyaatu jajabeesun nibarbaachisa |  |  |  |
| Daa’ima kiya Yeroo nyaachisu ija keesa laala haasofsiisa nyaachisa |  |  |  |

***Galatooma***

***Hora bulaa***
