# Supplementary material for: Magnitude and factors associated with appropriate complementary feeding practice among mothers of children 6–23 months age in Shashemene town, Oromia- Ethiopia: Community based cross sectional study
Source: PLoS One. 2022 Mar 29;17(3):e0265716. doi: 10.1371/journal.pone.0265716 (PMC8963544; doi:10.1371/journal.pone.0265716)
Supplement: S1 File — (ZIP) [file pone.0265716.s001.zip › supporting information/questionnrrie 11.pdf]

ANNEXI:

Participant informationsheet andinformed consent form

My Name is--------------------------. I am working as a data collector for the study being conducted on Magnitude and Factors associated with appropriate complementary feeding practice among mothers having children 6–23 months of age in Shashemene Town, Oromia, Ethiopia. , south Ethiopia,2020.by Junayidi Abdurahaman and kebede kumsa, who is teaching in Madda walabu University. I kindly request you to give me your attention to explain you about the study and being selected as a study participant.

The study title:

Magnitude and Factors associated with appropriate complementary feeding practice among mothers having children 6–23 months of age in Shashemene Town, Oromia, Ethiopia.

Purpose of the study:

The findings of this study will be used to identify The objective of this study was to assess the prevalence and associated factors with appropriate complementary feeding practice among mothers having children 6-23 months . The result of this study will contribute evidence and input for the district and as well as Zonal health office and other non-governmental organizations to address and solve these problems.

Procedure and duration:

You are selected randomly and we are inviting you to take part in the study. Your participation will help us to identify assess the prevalence and associated factors with appropriate complementary feeding practice among mothers having children 6-23 months We are going to ask you questions. Your honest answers are very useful for our study.The interview will take 10-15 minutes, so we kindly request you to give us the time for the interview. We would like to appreciate your help in responding to these questions.

Risks and benefits:

The risk of participiating in this study is very minimal, but only taking 10-15 minutes from your time. There is no direct payment for participating in this study. But the findings from this research may reveal important information for the district health office and Zonal health office and those concerned body.

Confidenentiality:

The information you will provide us will be confidential. There will be no information that will identify in particular. The findings of the study will be general for the studycommunity and will not reflect any thing particularly of individual persons. The questinniare will becoded to exclude showing names.

Rights:

Participation for this study is fully voluntary. You have the right to declare to participate or not in this study. If you decide to participate,you have the right to withdrawfrom thestudy at any time and this will notlabel you for any loss of benefits which you otherwiseare entitled.

Contact address:

If there are any questions or enquires about the study or procedures, please contact by this address at any time.

Principal investigator

Kebede kumsa Mobile phone -0926117614

Email-kebedekumsa929@gmail.com

Co-Investigators:

Junayidi Abdurahaman (BSc, MPH)-

Consent statement to be filled by participants

It was read to me the participant information sheet. I have clearly understood the purpose of the research,the procedures, the risks and benefites, issues of confidentiality,the right of participation. I have been given the opportunity to ask any questions for things that may have been unclear. I was informed that I have the right to withdraw from the study at any time. I have agreed to participate in the study.

A. Yes……. B. No……

Signature of the participant---------------- Signature of data collector----------------------------

	**Code ------- local area (kebele) ------------- Date data collected--------**
1	Age of mother
2	Educational status of mother<br>1.basic education 2.primary education<br>3.secondary education 4.higher education
3	Ethnicity of mother 1.oromo 2.Amahara 3.wolayita 4.tigree 9.others
4	Occupation of mother<br>1. House wife 2.house worker 3.gvt. employe 4 student 5.marchant 9.other
5	Religion of mother 1.musilim 2.orthodox 3.catholoc 4.protestant 9.other
6	Age at current delivery______________
7	Educational status of father<br>1.basic education 2.primary education<br>3.secondary education 4.higher education
8	Marital status of the mother<br>1. Married 2.not married 3. Divorced 4.widowed 5. Live at different place
9.	Occupation of father<br>1. Gov. worker 2.farmer 3.marchant 9.other
10.	Head of the house hold 1.male 2.female
11.	Monthly income of house hold per month______________
12.	Types of food preferred 1. Animal sources 2. Cereals
13.	Have you ever pregnant even if baby is not alive? 1. Yes 2. No
14.	No Pregnancy
15.	Birth Order of last delivered child aged (6-23Mo) 1. 1st 2. 2nd 3.3rd 4.4th 5.5th &above
16.	Are you attended ANC service follow up 1.yes 2.no
17.	No of ANC follow up visit attended?
18.	When did you started ANC follow up visit? 1.1-3 mo 2.4-5 mo 3.6 and above month
19.	How many children you delivered?

20.	Where did you delivered? 1.home 2. Health facility

21	Sex of the child? 1.male 2.female
22	Age of the child in month? describe it
23	Who assisted you to deliver 1.health professional 2.traditional attendants 9.other
24.	Is the delivery is planned?
25	Mode of delivery? 1. Normal 2.C/S 9.other
26	Birth interval?
27	Are you attended post natal care? 1.yes 2.no
28	If you attended post natal care when? 1.0-2 day 2. 3-6 day 3.7 and above
28.B	Have you ever breast feed your child ? 1. yes 2. No
29 A	After delivery when you give the first breast milk? 1. Immediately after birth 2.within one hour 3. within 24 hour 9.other
29B.	How long you gave Breat Milk for the baby?
30.	Are feed Breast Milk based on child demand by increasing frequency for 2and above year? 1. Yes 2. No
**Complementary feeding (Introduction of solid, semisolid or soft foods)**	
31.	In the last 24 hours, did the child taken foods such as solid,semisolid or soft foods? 1.yes 2.no
32.	If yes, what does the child taken? Describe it?
33.	How many times did a child eat solid, semisolid, or soft foods other than liquids yesterday during the day or at night? Please describe everything that child ate yesterday during the day or night, whether at home or outside the home
34.	Did child drink anything from a bottle with a nipple yesterday during the day or night?<br>yes .... 1 no .... 2 don’t Know --------8
35.	Did the child had diarrhea? 1. Yes 2. No
36.	Child had ARI (in last 2 weeks)? 1. Yes 2. No
37.	Did the child have a fever in the last time? 1.yes 2.no
38.	Complementary feeding practices and frequency during illness<br>1.Decrease quantity and frequency of food during illness<br>2. Withheld quantity and frequency of food during illness<br>3. Maintain same quantity and frequency during illness<br>4. Increase food quantity and frequency during illness
39.	Is there cultural and social food restriction? 1.yes 2.no
40.	If yes what is it?

41.	Mother's preferences about preparation of complementary foods? 1.Prepare separately CF for children 2. Prepare combine as an adult food
42.	Preferences about complementary foods? 1. Home- made 2. Commercially available foods
43.	Preferred food in complementary feeding? 1. Bread 2. Vegetables 3. Cow milk 4. Fruits / juices
	**Minimum dietary diversity**
	Probe Types of food given yesterday day and night the child 6–23 months [Minimum for dietary diversity /24 h] more than one answer is possibl**e**<br>45.Grains, roots and tubers 1. Yes 2. No<br>46. Legumes and nuts 1. Yes 2. No<br>47. Dairy products 1. Yes 2. No<br>48. Flesh foods (meat of hen, goat, sheep, cow) 1. Yes 2. No<br>49. Fish meat 1. Yes 2. No<br>50. Eggs 1. Yes 2. No<br>51. pumpkin, carrots, sweat potatoes 1. Yes 2. No<br>52. Potatoes, cassava, roots 1. Yes 2. No<br>53. Dark green leafy vegetable 1. Yes 2. No<br>54. Other fruits and vegetable 1. Yes 2. No
55.	Did the child taken organ meats such as kidney ,liver and hearts? 1. Yes 2. No
56.	Did the child had consumed foods prepared from oil/butter? 1. Yes 2. No
57.	Did the child had consumed sweat foods like ’’chocolates, sweets, candies, pastries, cakes, or biscuits ‘?1. Yes 2. No
58.	Did the child had consumed foods such as (Ripe mangoes, ripe papayas tomatoes, and green pepper) Vitamin A-rich fruits and vegetables 1. Yes 2. No
59.	Did the child have consumed Other fruits and vegetable one day before the survay (‘fruits and vegetables? 1. Yes 2. No

60.	Who decide on properties of house hold? 1.husband 2.wife 3.jointly
61.	Where did you get information about advantage of breast feeding? 1. TV 2. Radio 3. Reading 4. Health professional. 9. Other (specify)?
62.	Where did you get information about source commercially prepared complementary food?<br>1.health care worker 2.family 3.media 4.relative
63.	Do you have knowledge about the advantage of iron? 1.yes 2.no
64.	Do you know the benefit of Iodized salt? 1.yes 2.no
	**(Minimum meal frequency (MMF)**
65	Minimum meal frequency given for the child Breast fed ?
66	Yesterday at night or day, did the child consumed breast milk of other person by cup/spoon?
67	Minimum meal frequency given for the child non Breast fed?
68	For breast feed child aged (6-23) did he/she received solid, semi-solid, or soft foods during the previous day? 1.yes 2.no
69	For not breast feed child aged (6-23) did he/she received solid, semi-solid/ soft foods including milk during the previous day?
70	Write Months /Time of initiation of complementary feeding?
71	Reason for early/late initiation of complementary food?
74	Water source of house hold? 1.pipe /bono 2.ground water using pipe 3.manually prepared ground water 4.stream water 5.collected rain water 6.surface water(rive, pond ,stream, dam)<br>7.factory prepared food 8.other
75	House in which you live 1.self-owned 2.rented
76	Type of House hold toilet?<br>1. Ventilated improved latrine 2. Has seat slab 3.has no seat slab<br>4. Used for composting purpose 5. Made from bucket for urination or defecation<br>6. Hanging toilet 7.no latrine use open defecation 9. Other (specify)

	**House construction**

77.	Floor of natural house? 1. Soil 2. Mad 3. Lumber 4. Bamboo tree
78	The base of inside house is made of ?<br>1. lumbered wood 2. ceramic tiles 3.cement 4.other (specify)
79	Roof of the house is made from? 1. Corrugated sheet of iron 2. grass 3.Solil on the roof
80.	The base of the house is made of? 1. Metal 2. Wood/bamboo tree 3.other(specify)
81	Base establishment of house is? 1.bamboo with mad 2.stone with mad 3.blocket<br>4. komborsato cardboard, play wood 5. reused wood
82	Finished wall made of?<br>1. Cement<br>2. Stone with cement<br>3. Bricks<br>4. Bloket made of mad /Covered adobe/<br>5. Wood planks 9. Other (specify)
83	No of rooms of house hold? 1.one room 2.two room 3.more than two room
84	Number of rooms for sleeping ?
85	Ask Whether a household or one of its members owns the following items **more than** one ans. is possible<br>1. access to electric city 1.yes 2.no<br>2. radio 1.yes 2.no<br>3. TV 1.yes 2.no<br>4. phone 1.yes 2.no<br>5. Refrigerator 1.yes 2.no<br>6.chair 1.yes 2.no<br>7. bed 1.yes 2.no<br>8. Electric stove 1.yes 2.no<br>9 kerosene 1.yes 2.no<br>10.lamp 1.yes 2.no<br>11.watch 1.yes 2.no<br>12.mobile phone 1.yes 2.no<br>If less 1 write “00” if more than one write “ 95 “ if not known write “99”

Household Food Insecurity Access Scale (HFIAS)			
	**Occurrence Questions**	Ans. No(skip to next Q)	How often?
1	In the past 4 weeks, did you worry that your household would not have enough food?	1.Yes 2.No	1.Rarely 2.sometimes 3.often
2	In the past 4 weeks, were you or any household member not able to eat the kinds of foods you preferred because of a lack of resources?	1.Yes 2.No	1.Rarely 2.sometimes 3.often
3	In the past 4 weeks, did you or any household member have to eat a limited variety of foods due to a lack of resources?	1.Yes 2.No	1.Rarely 2.sometimes 3.often
4.	In the past 4 weeks, did you or any household member have to eat some foods that you really did not want to eat because of a lack of resources to obtain other types of food?	1.Yes 2.No	1.Rarely 2.sometimes 3.often
5	In the past 4 weeks, did you or any household member have to eat a smaller meal than you felt you needed because there was not enough food?	1.Yes 2.No	1.Rarely 2.sometimes 3.often
6	In the past 4 weeks, did you or any household member have to eat fewer meals in a day because there was not enough food?	1.Yes 2.No	1.Rarely 2.sometimes 3.often
7	In the past 4 weeks, was there ever no food to eat of any kind in your household because of lack of resources to get food?	1.Yes 2.No	1.Rarely 2.sometimes 3.often
8	In the past 4weeks, did you or any household member go to sleep at night hungry because there was not enough food?	1.Yes 2.No	1.Rarely 2.sometimes 3.often
9	In the past 4 weeks, did you or any household member go a whole day and night without eating anything because there was not enough food?	1.Yes 2.No	1.Rarely 2.sometimes 3.often

Next I would like to ask you about some liquids that ***<u>(NAME)</u>*** may have had yesterday during the day or at night.			
No	Type of food	CODING CATEGORIES	Frequency consuming
1.	Plain water? 1 yes 2.No 3.Dnot Know	Yes 1 No 2	
2.	Infant formula such as [**insert local examples**]?	Yes 1 No 2	
3.	Milk such as tinned, powdered, or fresh animal milk?	Yes 1 No 2	
4.	Juice or juice drinks?	Yes 1 No 2	
5.	Clear broth?	Yes 1 No 2	
6.	Yogurt?	Yes 1 No 2	
7.	Thin porridge?	Yes 1 No 2	
8.	Any other liquids such as [list other water-based liquids available in the local setting]?	Yes 1 No 2	

**Mothers attitude question**	Level of education		Age	
	1^{ry}	2^{ry}		
It is important to help my child when she eat				
It is important to feed my child to eat slowly and patiently.				
It is important to encourage my child to eat				
I talk to my child during feeding by looking straight in the eyes				
