# Supplementary material for: Magnitude and factors associated with appropriate complementary feeding practice among mothers of children 6–23 months age in Shashemene town, Oromia- Ethiopia: Community based cross sectional study
Source: PLoS One. 2022 Mar 29;17(3):e0265716. doi: 10.1371/journal.pone.0265716 (PMC8963544; doi:10.1371/journal.pone.0265716)
Supplement: Attachment — Submitted filename: response to revewier from aouthers1.pdf [file pone.0265716.s003.pdf]

Response to each point raised by the academic editor, and reviewer.

Dear/Sir, I hope this message find you well. Thanks very much for your very valuable and constructive comments that you input to our manuscript. We have deeply appreciate for your grate contribution in providing us the knowledge, experiences you have and you precious time that you invests to look at the manuscript line by line "thanks o much more again". Upon checking your comments we found that majority of the comments were very helpful for improving our manuscript so that we addressed as per the provided comments/suggestions. Regarding each response to reviewer(s) the responses to comments were stated below as follow.	
**Reviewer comments**	**Response from Author(s)**
1. Please ensure that your manuscript meets PLOS ONE's style requirements, including those for file naming. The PLOS ONE style templates can be found at	Thanks for your valuable comments<br>We have checked and ensured that our manuscript meets PLOS ONE's style requirements
Comment (A2) Why targeting appropriate practices if inappropriate practices are the problem to solve?	**Answer**: Thanks for your Question. You are quit right. But to differentiate b/n wrong and right, bad and good, legal and illegal, appropriate and inappropriate it's better to know the benchmark/ set of criteria then try to compare it against the dictated criteria.so ,in order to solve the inappropriate our finding was categorized as appropriate Vs inappropriate then act on the inappropriate one. plus appropriate feeding practices are critical for improving nutritional status and ensuring child survival which helps to know the gap in intervention**[please see line 24-25]**
Comment (A3). In this part, you shall indicate shortly data collection method and main indicators used in the Paper.	Thanks for comments. we add the following "Data was collected by Face-to-face interviews during home-to-home visits with mothers who had children aged 6–23 months, using a structured questionnaire on the main complementary feeding indicators". **Would you see line 30-32,look under the Abstract Method**
2. Please provide additional details regarding participant consent. In the ethics statement in the Methods and online submission information, please ensure that you have specified what type you obtained (for instance, written or verbal, and if verbal, how it was documented and witnessed). If your study included minors, state whether you obtained consent from parents or guardians. If the need for consent was waived by the ethics committee, please include this information.	**Answer**: Thank you for your helpful feedback. As you mentioned, we rewrite the method section and added: we add the following points 'before we collected data from each individual, information about informed verbal consent was provided then the informed consent was obtained from parent or guardian''. Plus we stated in the document that ethical issues were in line with the ethical principle of "autonomy" statements that give participants the right to stop participation in the study. **Answer** we add this in the manuscript ' In short, the information sheets were available on each questionnaire then, we provided information about the purpose, procedures, benefits and disadvantage of the study for the participants or guardians. Informed verbal consent was obtained from each study participants due

	to the nature of the study was not invasive. Lastly, only participants who were willing to participate in the study were included. Moreover, those who wish to stop their participation at any stage were also permissible to do so without any restriction'. Would you look at manuscript with track change? **Please see line 255-268**
3. Please include additional information regarding the survey or questionnaire used in the study and ensure that you have provided sufficient details that others could replicate the analyses. For instance, if you developed a questionnaire as part of this study and it is not under a copyright more restrictive than CC-BY, please include a copy, in both the original language and English, as Supporting Information. If the original language is written in non-Latin characters, for example Amharic, Chinese, or Korean, please use a file format that ensures these characters are visible.	**Answer**: Thanks for your nice suggestions. Just as the reviewers comments we included the questionnaire in supporting information and the source of questionnaires was stated in the manuscript. It was developed by reviewing different literature and documents like [5,6] Infant and young child feeding indicators, and Adapted to our country's context. Would you see the reference [5.6.13, 14]. **would you see manuscript with tack change line 216-235**
4. During your revisions, please confirm whether the wording in the title is correct and update it in the manuscript file and online submission information if needed. Specifically, please correct the title on the pdf.	**Answer**: Thanks for reminding key issue on the topic we did as suggested by the reviewer. It was corrected.<br>Magnitude And Factors Associated With Appropriate Complementary Feeding Practice Among Mothers Having Children 6–23 Months Of Age In Shashemene Town, Oromia- Ethiopia: Community Based Cross Sectional Study**.this is the first**
Magnitude and Factors associated with appropriate complementary feeding practice among mothers having children 6–23 months of age in Shashemene Town, Oromia- Ethiopia: community based cross sectional study.	Magnitude And Factors Associated With Appropriate Complementary Feeding Practice Among Mothers of Children 6–23 Months Age in Shashemene Town, Oromia- Ethiopia: Community Based Cross Sectional Study**. The updated topic would you see line 2-5**
5. Please update your submission to use the PLOS LaTeX template. The template and more information on our requirements for LaTeX submissions can be found at http://journals.plos.org/plosone/s/latex.	**Answer**: Thanks for your comments .we have check and assured as the manuscript meets the PLOS template.
6. Thank you for stating the following financial disclosure:<br>"The funders had no role in study design, data collection and	**Answer**: Thank you for your suggestion; We did this research as our employer

analysis, decision to publish, or preparation of the manuscript."<br><br>At this time, please address the following queries:<br><br>a) Please clarify the sources of funding (financial or material support) for your study. List the grants or organizations that supported your study, including funding received from your institution.<br>b) State what role the funders took in the study. If the funders had no role in your study, please state: "The funders had no role in study design, data collection and analysis, decision to publish, or preparation of the manuscript."<br>c) If any authors received a salary from any of your funders, please state which authors and which funders.<br>d) If you did not receive any funding for this study, please state: "The authors received no specific funding for this work."Please include your amended statements within your cover letter; we will change the online submission form on your behalf.	routine duty. Regarding the organization we thank Madda Walabu university Shashemene Campus for their indispensible support and funding to undertake this research. As it was mentioned in the document body the funders had no role in study design, data collection and analysis, decision to publish, or preparation of the manuscript." However, Madda walabu university funded us with funding letter reference number **RMWU-14/84/355.** But **the funding was not for publication.**<br><br>To be genuine, all of the authors received a salary from the funder hence all are the employee of the University. We are extremely sorry, since we did not write the reference number on system so would you accept our apology for that? We updated it. It was stated in cover letter, too. [**would you see line 621-625 and cover page about authors**]
7. Thank you for stating the following in the Acknowledgments Section of your manuscript:<br><br>"The investigators would like to thanks Madda Walabu University, Shashemene Campus for offering the fund to carry out this research project that is targeted to find out community problems. Materials used in this project are honorable acknowledged. The investigators express<br>their sincere appreciations to peoples and organization (Shashemene Health Bureau, Shashemene City Administration for offering necessary information that help to conduct the research. Lastly, we would like to present our heartfelt thanks to data collectors and study participant for support they provided us throughout the whole process of data collection period. "<br><br>We note that you have provided funding information that is not currently declared in your Funding Statement. However, funding information should not appear in the Acknowledgments section or other areas of your manuscript. We will only publish funding information present in the Funding Statement section of the online submission form.<br>Please remove any funding-related text from the manuscript and let us know how you would like to update your Funding	**Answer**: Thanks for your kind information that provide Us a lot about pols style of writing, as you recommendation we removed funding related text written in the acknowledgement part of the manuscript. [**would you see line 621-625 and cover page about authors]**<br><br>Regarding the materials mentioned in **acknowledgement** part we mean that the references or documents that used for developing the proposal or this manuscript.

Statement. Currently, your Funding Statement reads as follows:<br><br>"The investigators would like to thanks Madda Walabu University, Shashemene Campus for offering the fund to carry out this research project that is targeted to find out community problems."<br><br>Please include your amended statements within your cover letter; we will change the online submission form on your behalf	
8. In your Data Availability statement, you have not specified where the minimal data set underlying the results described in your manuscript can be found. PLOS defines a study's minimal data set as the underlying data used to reach the conclusions drawn in the manuscript and any additional data required to replicate the reported study findings in their entirety. All PLOS journals require that the minimal data set be made fully available. For more information about our data policy, please see http://journals.plos.org/plosone/s/data-availability.<br><br>Upon re-submitting your revised manuscript, please upload your study's minimal underlying data set as either Supporting Information files or to a stable, public repository and include the relevant URLs, DOIs, or accession numbers within your revised cover letter. For a list of acceptable repositories, please see http://journals.plos.org/plosone/s/data-availability#loc-recommended-repositories. Any potentially identifying patient information must be fully anonymized.<br><br>Important: If there are ethical or legal restrictions to sharing your data publicly, please explain these restrictions in detail. Please see our guidelines for more information on what we consider unacceptable restrictions to publicly sharing data: http://journals.plos.org/plosone/s/data-availability#loc-unacceptable-data-access-restrictions. Note that it is not acceptable for the authors to be the sole named individuals responsible for ensuring data access.<br><br>We will update your Data Availability statement to reflect the information you provide in your cover letter.	**Answer**: Data set underlying the results described in our manuscript beside this, the detail of the data also available in supporting information without any restriction.<br>In short, We confirmed that all data underlying the findings described in this manuscript are fully available without restriction i.e 'No restriction will apply'
9. PLOS requires an ORCID iD for the corresponding author in Editorial Manager on papers submitted after December 6th, 2016. Please ensure that you have an ORCID iD and that it is validated	**Answer** : We have valid orchid ID for your information 0000-0002-7783-7883

in Editorial Manager. To do this, go to 'Update my Information' (in the upper left-hand corner of the main menu), and click on the Fetch/Validate link next to the ORCID field. This will take you to the ORCID site and allow you to create a new iD or authenticate a pre-existing iD in Editorial Manager. Please see the following video for instructions on linking an ORCID iD to your Editorial Manager account: https://www.youtube.com/watch?v=_xcclfuvtxQ	
10. Please ensure that you refer to Figure 2 in your text as, if accepted, production will need this reference to link the reader to the figure.	**Answer** : Thank you for your suggestion .We are very sorry, really it was a mistake we made, accordingly it was corrected and we have updated thus the information was in the revised manuscript. (**See page 8 line 215 of the manuscript with track changes**)
11. "Please upload a copy of Figure 4, to which you refer in your text on page 15. If the figure is no longer to be included as parts of the submission please remove all reference to it within the text.	**Answer**: Thank you for your valid and constructive comment or information and we are very sorry for missing upload of very important data.it was updated as per your suggestion. **You may check on the system.**
12. Thank you for submitting the above manuscript to PLOS ONE. During our internal evaluation of the manuscript, we found significant text overlap between your submission and the following previously published works, some of which you are an author.<br><br>- https://bmcnutr.biomedcentral.com/articles/10.1186/s40795-017-0202-y<br>- https://bmcpediatr.biomedcentral.com/articles/10.1186/s12887-016-0675-x<br>- https://www.hindawi.com/journals/jnme/2019/2869424/<br><br>We would like to make you aware that copying extracts from previous publications, especially outside the methods section, word-for-word is unacceptable. In addition, the reproduction of text from published reports has implications for the copyright that may apply to the publications.<br><br>Please revise the manuscript to rephrase the duplicated text, cite your sources, and provide details as to how the current manuscript advances on previous work. Please note that further consideration is dependent on the submission of a manuscript that addresses these concerns about the overlap in text with published work.	**Answer**: We have special concern for your valuable suggestion and Thanks for that still we did our best to rephrase it carefully in all parts of document beginning from the title to the Reference.

We will carefully review your manuscript upon resubmission, so please ensure that your revision is thorough.	
1) Please avoid using abbreviations without explanation in the abstract. Always define abbreviations at first mention.	**Answer**: We appreciate your comments, so it was corrected as per the reviewer (s) suggestions for example in abstract. **would you see line 31 and 38**
2) Please provide global or country wide estimate of complementary feeding practice	**Answer**: Introduction line (53-63) Malnutrition is still one of the leading causes of morbidity and mortality among children worldwide. It has been directly or indirectly responsible for 60 percent of the 10.9 million deaths among children under the age of five each year, with two-thirds of these deaths frequently associated with inappropriate feeding practices [1, 2].<br><br>As indicated in line introduction paragraph 3 lines (60-73) Inadequate complementary feeding practices have been identified as a global risk factor for diarrheal diseases, malnutrition outcomes, and under-5 mortality [7.]. Only one in every six children worldwide receives a minimally acceptable diet [8]. According to a study conducted in South Asia, there are significant differences in complementary feeding practices. For example, India had the lowest proportion 15% of children 15% who met the minimum dietary diversity (MDD), followed by Nepal (34%), Bangladesh (42%), and Sri Lanka (71%) [9, 10]. One out of every ten infants and young children received minimum acceptable diet in Eastern and Southern Africa [8]. According to the EDHS (2016)[11], only 7% of children aged 6-23 months met the criteria for a minimum acceptable diet, the lowest ever; Ethiopia has taken various steps in the implementation of the Integrated Child Development Scheme to improve children's nutritional status [12]. However, evidence suggests that appropriate complementary feeding practices are still low. According to a study conducted in Nagele Arsi, the prevalence of appropriate complementary feeding practices was 9.5 percent, the minimum acceptable diet was 12.3 percent [13] and Damota sore was 11.4 percent [14].

3) Why Ethiopia? "Ethiopia is one of the sub Saharan African countries with high level of malnutrition and has launched the national strategy for infant and young child feeding in 2004 to improve the nutritional status of children". This is all that is written about Ethiopia in the introduction which is not enough reason to conduct a study on complementary feeding in Ethiopia.	Answer: Thanks so much for your useful feedback about Ethiopia on introduction. we were add the following points "<br>**Introduction paragraph 3 line (66-80)** "According to EDHS (2016) only 7 % of children age 6-23 months have met the criteria for a minimum acceptable diet this is the lowest compared to that of India 9%, Sri Lankan 68%, Bangladeshi 40% and Nepal 32% children [(9,10)]. Although this is a very vulnerable period and It is also the time when malnutrition starts in many infants, contributing to the high prevalence of malnutrition in children under two years of age.<br><br>Malnutrition is global concern to meet SDGs thus; Ethiopia has taken various steps in implementation of the Integrated Child Development Scheme [12], to improve the nutritional status of children. But evidence suggests that appropriate complementary feeding practices remain low. As study done in Nagele Arsi the prevalence of appropriate complementary feeding practices was 9.5 %, Minimum acceptable diet was 12.3 % [13,] while that of Damota sore 11.4% [14].

4) Please provide a proper literature review on complementary feeding practice globally and in Ethiopia.<br><br>What does existing literature say about the prevalence and factors associated with complementary feeding practice? Why is it a topic of concern?	**Answer: would you see line 63-104.** For this question the answer was stated above however regarding the '**Why is it a topic of concern?**<br>Mothers' child feeding practice should be known in the study area hence child feeding practice can be changed with time with in short period of time and due to rapid urbanization. Malnutrition is global concern that linked with inappropriate feeding practice that can be preventable in this window of opportunities, children 6-23month age is a very vulnerable period and It is also the time when malnutrition starts in many infants, contributing to the high prevalence of malnutrition in children under two years of age.<br><br>**Please see line 81-86.** Concerning factors associated with appropriate complementary feeding practices of children aged 6–23 months Previous studies conducted elsewhere show higher maternal and paternal education, better household wealth, exposure to media, adequate antenatal and post-natal contacts, child's sex and age, institutional delivery, low parity, maternal occupation, urban residence, knowledge & frequency of complementary feeding and receiving feeding advice about immunization was determinant factors for appropriate complementary feeding[**9,10**].<br><br>Studies conducted so far in Ethiopia on appropriate complementary feeding practices were used single or three indicators to study appropriate complementary feeding for example may use only about exclusive breast feeding or used three indictors about CF. Ethiopia particularly, in Shashamene there were diversified

	ethnic groups that have different cultures ,beliefs or feeding habit or customs affects child feeding. Beside this, currently EBF practice were also reduced in Ethiopia as EDHS[2016].So, Knowing the level of practices helps to undertake appropriate measures to improve child feeding practice that in turn improve nutritional status of children through this essential time of child growth and development. so that the study was designed to find out the prevalence and factors associated with ACFP [5. , UNICEF. Programming Guide for Infant and Young Child Feeding, Nutrition Section Programmes. New York: UNICEF; 2011. WHO and UNICEF]. **Would you refer to line 84-104**
5) The buildup to the study rationale is weak.	Answer: Thanks for your helpful constructive comments I hope this was answered in the above question[Q4]
6) A proper definition of the independent variables would be appreciated. For instance, what do you mean by food insecurity? How is it derived?	Discussed under Methodology section in sub topic dependent and independent variable paragraph 1 line (134-188) - Household food security was measured with the Household Hunger Scale (HHS) which has 9 items along with 9 frequencies (9I 9F) Household Food Insecurity Access Scale (HFIAS). The household food insecurity status was measured by direct survey of household consumption 4weeks preceding the survey. In this study, household food insecurity is dichotomous variable taking value 1 if the household is food insecure and 0 otherwise.<br><br>The response categories are never (0 times), rarely (1–2 times), or sometimes (3–10 times), and often (more than 10 times). Therefore, the HHS was used in this study to define two groups; households reporting (a) **little to no hunger** in the past

	month because of insufficient food or because of lack of resources to get food and thereby classified as **food secure** households, and (b) **moderate to severe hunger** in the past month because of insufficient food or because of lack of resources to get food, and thereby classified as **food insecure** households. **Please would you refer to line 179-188**
7) How was the effect of residual confounding as a result of unmeasured co-variates handled?	**Thanks for your comments, Please see 245-248.** Residual confounding is the distortion that remains after controlling for confounding in the design and/or analysis of a study. The effect of residual confounding as a result of unmeasured co-variates handled with multivariate analyses using multiple logistic regression models to assess the presences of association, to identify and control of confounding variables. However, the effect of unmeasured or unknown confounders may only be controlled by true randomization in a study with a large sample size.<br>There are three causes of residual confounding:<br>1. There were additional confounding factors that were not considered, or there was no attempt to adjust for them, because data on these factors was not collected.<br>2. Control of confounding was not tight enough. For example, a study of the association between physical activity and age might control for confounding by age by a) restricting the study population to subject between the ages of 30-80 or b) matching subjects by age within 20 year categories. In either event there might be persistent differences in age among the groups being compared. Residual differences in confounding might also occur in a randomized clinical trial if the sample size was small.

	In a stratified analysis or in a regression analysis there could be residual confounding because data on confounding variable was not precise enough, e.g., age was simply classified as "young" or "old".<br>3. There were many errors in the classification of subjects with respect to confounding variables.<br><br>Even when all confounding factors are known and controlled for using conventional multivariate analysis, the observed association between exposure and outcome can still be dominated by residual confounding effects. Therefore, an observed significant association apparently provides limited evidence for a causal relationship. We explain this issue in the limitation part of the study as our study design was cross-sectional it is not exempted from problem cause effect relationship. To handle the effect of residual confounding effect we took representative sample size<br>Beside this we used standardized questionnaire to include all variable that are important to measure appropriate complementary feeding as well we tried to control confounding with multivariate analysis.
8) How was the dependent variable expressed in the analysis?	**Answers**: Thanks for your concern, the dependent Variable was expressed as: Mothers' complementary feeding practices which the response can be dichotomized and coded as [1= Appropriate complementary feeding practice, 0= inappropriate complementary feeding practice] ,Appropriate complementary feeding practice: --defined appropriate when they meet all the four CF indicators timely introduction, minimum meal frequency and minimum dietary diversity and minimum acceptable diet and coded as 1 while it is considered inappropriate complementary feeding practice when it fails to fulfill even a single indicator (5).**Please would you see line 134-173**

9) How was statistical bias account for and avoided?	Statistical bias is anything that leads to a systematic difference between the true parameters of a population and the statistics used to estimate those parameters. In other words. In research, bias occurs when "systematic error is introduced into sampling or testing by selecting or encouraging one outcome or answer over others". Bias can occur at any phase of research, including study design or data collection methods, as well as in the process of data analysis and publication.<br><br>Bias cannot usually be totally eliminated and some degree of bias is nearly always present in a published study so; the aim is to keep it to a minimum, to identify those biases that cannot be avoided. The motto of epidemiologist could well be "dirty hand but clean mind" [manus sordidae, mens pura].**Therefore, knowing different types of bias, when they occur, and how they might be avoided is very important for example some common types of sampling bias include self - selection, non-response, under coverage, survivorship, pre-screening or advertising, and health user bias.**<br><br>**Sampling bias in probability samples**<br><br>In probability sampling, every member of the population has a known chance of being selected. For instance, you can use a random number generator to select a simple random sample from your population. Although this procedure reduces the risk of sampling bias, it may not eliminate it. If your sampling frame the actual list of individuals that the sample is drawn from does not match the population, this can result in a biased sample.so, **using carful research design and sampling procedures** can help to **avoid sampling bias**. **Oversampling** can be used to correct under coverage. Chance and confounding can be quantified and/or eliminated through proper study design and data analysis. **However, only the most rigorously conducted trials can completely exclude bias as an alternate explanation for an association.** Unlike random error, which results from sampling variability and which decreases as sample size increases.<br><br>So, reviewers of the literature must consider the degree to which bias was

	prevented by **proper study design and implementation**. To sum up Bias can occur in the planning, data collection, analysis, and publication phases of research.so careful, proper design and data analysis procedures can help to avoid sapling bias. [Christopher J. Pannucci, MD and Edwin G. Wilkins, MD MS University of Michigan Ann Arbor, Michigan.<br><br>Identifying and avoiding bias in research, NIH Public Access Author Manuscript Published in final edited form as: Plast Reconstr Surg Author manuscript; available in PMC 2011. 2010August; 126(2):619–625. doi:10.1097/PRS.0b013e3181de24bc]
Comment 2: Why targeting appropriate practices if inappropriate practices are the problem to solve?	**Answer**: Thanks for valuable comments appropriate feeding practices are critical for improving nutritional status and ensuring child survival which helps to know the gap in intervention[**please see line 24-24].it was answered above.**
Comment 3 in this part ,you shall indicate shortly data collection methods and main indicators used in the paper	**Answer:** Data was collected by Face-to-face interviews during home-to-home visits with mothers who had children aged 6–23 months, using a structured questionnaire on the main complementary feeding indicators. [These were introduction to solid or soft food, MMF, MDD, MAD]. **please see line 30-32**
Comment 4 use the recommended désignation	Thanks for your comment was addressed as per the suggestion given minimum dietary diversity, minimum acceptable diet etc. **look at under the abstract part please. line 36-39**
Comment 5 What it is this? Is it a new indicator?	No, it is not new, appropriate complementary feeding practices are a measure that helps to measure the level mothers' infant and child feeding practices that incorporate [all the four Complementary feeding indicators timely introduction, minimum meal frequency and minimum dietary diversity and minimum acceptable die. would look at line 137-140
Defline at the first occurrence (ANC=anténatal care)	It was defined as the health care services given to the pregnant mothers during the pregnancy for targeting to ensure the health of the mother and the baby via screening

	for complication **would you see line 293-294**
Comment 7 indicators	As per the comment indicators were defined in the methods section as well in the abstract part. Would you see line 137-140we add" **Appropriate complementary feeding practice**: defined appropriate when they meet all the four Complementary feeding indicators timely introduction, minimum meal frequency and minimum dietary diversity and minimum acceptable diet and coded as 1 while it is considered inappropriate complementary feeding practice when it fails to fulfill even a single indicator [5,6].<br>**Timely introduction of complementary feeding**: The proportion of children 6–23 months that were introduced to solid and semisolid foods at 6 months of age **[5,6]**.<br><br>**Minimum dietary diversity:** is the proportion of children 6–23 months of age who receive foods from 4 or more food groups with the food groups consisting; (I) grains, roots and tubers; (II) legumes and nuts; (III) dairy products; (IV) flesh foods; (V) eggs; (VI) vitamin A rich fruits and vegetables; and (vii) other fruits and vegetables during the previous day of study **[5,6]**. **Minimum meal frequency:** is the proportion of breastfed and non-breastfed children 6–23 months of age, who receive solid, semi-solid, or soft foods (but also including milk feeds for non-breastfed children) the minimum number of times or more during the previous day.<br><br>**Minimum** is defined as 2 times for breastfed infants 6–8 months, 3 times for breastfed children 9–23 months, 4 times for non-breastfed children 6–23 months **[5,6]**.<br><br>**Minimum acceptable diet:** is the proportion of children 6–23 months of age who receive both minimum meal frequency and minimum dietary diversity during the

	previous day of study **[5,6]**.
Comment 10	The comments were addressed in detail in the introductory part. **Would you refer line 135-170**
Comment 11 Harmoniez 6-23/6-24months	It was harmonized as 6-23 months throughout the document
Comment (12) Thèses recommandations have been updated recently. Indicators for assessing infant and young child feeding practices: definitions and measurement methods (who.int)	Thank you for informing us the updated information about IYCF indicator we already used the former one developed in 2008, 2010.
Comment 14 &16, Please precise the region: country at least	According to a study conducted in Nagele Arsi Oromia Region ,Ethiopia the prevalence of appropriate complementary feeding practices was 9.5 percent, the minimum acceptable diet was 12.3 percent [13] and Damota sore of south Ethiopia was 11.4 percent [14].**would see line 74-76**
Comment 13	Thanks for your comments, we addressed in detail in introduction part. **Would you see line 93-107.**
Comment 15	Appropriate complementary feeding practice indicator **used were t**imely introduction of complementary feeding, Minimum meal frequency, Minimum dietary diversity, & Minimum acceptable diet. defined under methodology part. **Please see line 141-171.**
Comment 17 Rephrase; difficult to Under stand	It was rephrased look **at line 73-76**
Comment on the objective	The comments was addressed the objective of this study was to assess the prevalence of appropriate complementary feeding practices among mothers having 6-23 months children, from Feb.-march 2020 and associated factors in Shashemene Town. **would you see line 25-27**
Comment 18,19 : Add reference	Thanks for constructive comments; it was corrected as the suggestion. **would see line 140-154**

Comment (A22-23) add reference	Thanks for your comment, we Added 'Oromiya Regional State, Shashemene City Administration, Physical and Socio - Economic Profile Report. 2015 – 2016 G.C.' **would you see please line 664-665,The Reference N$^{\underline{o}}$18 line 669**
Mothers who resided in the study area for <6 months were excluded from the study subjects.	Thanks for your comment, we Added asper suggestion. **would you see line 128**
Comment A24 Repetition of the previous part. Suggest to delete	Thanks for your suggestions it was corrected asper recommended by the reviewer deleted from line133, 178. **would you see line 133,**
Comment on sampling	Thanks for your constructive comment we add as it was commented. The following assumptions were used:<br>- Margin of error=4%;<br>- Zα =1.96 and<br>- Design effect =2.<br>243 samples were obtained with consideration of 10% contingency to non-responders a total of 536 mothers were sampled .would you refer to line 193-199
Who collects data	Thanks for your feedback .Six diploma holder nurses corrected and stated under methods part. would you refer to line 226
Why this percentage5%?	Thanks for your feedback it was corrected as 'pre-tested 26 mothers'. would you refer to line 230
Formatted comments	Thanks for your feedback it was corrected asper the comments. would you refer to line237-241,
Comments in Data pressing and analysis	Thanks for your valuable contrition the comments on formatting was addressed accordingly.**it was indicated in line 237-251.would you see it?**
Comment (A30) ,MWU define it	**The Madda Walabu University (MWU) would see line 256?**

Comment (A31) how far ethical considérations?	As our view if problem was observed after analysis ,it is the issue of moral to develop proposal and act on unless we are neglecting them hence they had no information.
Comment (A33)Why did others not participate?	Thanks for your question; the reason is because they are not at home during the study data collection period.
Commets about the result part	All the comments are very important and constructive thus it was addressed asper the suggestions given for us.
Comment (A38) All the variables presented in this table should be presented in the methodology and the categorization and processing methods clearly presented	Thanks for your comments. All data presented were found in method part. Look at under the methods part. Please refer to line 134-189
Comment (A39),The total of the percentage must give 100	The total of the percentage was corrected, it was 100% now.it was addition error **62.5,18.3 ,15.5 ,2.5 ,1.2** very sorry for that , **please would you see table one**
Comment(A41)Please define	Thanks for your comments ;It was defined as Ethiopian Birr(ETB),would you see in the table and under the table **page 13,21.line 407-408**
Comment(A42)how accessed food insecurity	Thanks for your concern it is important Question. we assessed using 9 items along with 9 frequencies '(9I 9F)' **would mind looking at line 179-188**
Comment(A43) use only percentage in the text	We corrected as follow; The majority of study participants (67.5 percent) were multiparous, and (95 percent) received antenatal care. Four hundred fifty (86.5 percent) of respondents had a birth interval of more than
Comment (A44) define here or under the table 2	Thanks so much for your feedback. Abbreviations were defined in or under each tables as recommended by the reviewer(s), example TBAs=Traditional Birth Attendants, TBAs=Traditional Birth Attendants, HCW=health care workers<br>Please see Line 293-294, under the table 2
Comment (A46) present the Food group recommended	Minimum standards with respect to IYCF practices specifically, children age 6-23 months who have to meet all IYCF criteria below with of focus ;Timely, adequate & appropriate<br>1. Breast feeding, or not breastfeeding and receiving two or more feedings of commercial infant formula; fresh, tinned, or powdered animal milk; or yogurt.<br>2. Fed with foods from four or more of the following groups: **a**. infant

	formula, milk other than breast milk, and cheese, yogurt or other milk products; **b.** foods made from grains, roots, and tubers, including porridge and fortified baby food from grains; **c.** vitamin A-rich fruits and vegetables; **d.** other fruits and vegetables; **e**. eggs; **f.** meat, poultry, fish, and shellfish (and organ meats); and **g.** legumes and nuts.<br>3. Fed the minimum recommended number of times per day according to their age and Breastfeeding status [EDHS, (2016), UNICEF/WHO, (2008)].<br>Thanks for your kind suggestions; **would you See line 311-315**
Comment (A47) unclear 'while the rest 122(23.5%) was preferred commercially available food'	The proportion of mothers who preferred commercially available food for their children was 122 (23.5 percent). **please see line 316-317**
Commets (A48) about table 3	Nice suggestions thank a lot; we made change on it. **would you refer to line 332-333,or table 3**
Comments about Word/gram mars error	Many comments had been addressed in page 17, 18, 19, 20 thanks so much for such wonderful comments. **Please Would you see line 341-361,394-400**
Comment (A) Positively or negatively ?	In this study there are variables that positively associated with ACFP After controlling for potential confounders, like socioeconomic status (wealth index, household food security, and household income), sources of information, child age. the number of ANC follow-ups, mothers' knowledge, and positive attitude. **please see line 370-406,495-451**
