# Supplementary material for: Magnitude and factors associated with appropriate complementary feeding practice among mothers of children 6–23 months age in Shashemene town, Oromia- Ethiopia: Community based cross sectional study
Source: PLoS One. 2022 Mar 29;17(3):e0265716. doi: 10.1371/journal.pone.0265716 (PMC8963544; doi:10.1371/journal.pone.0265716)
Supplement: Attachment — Submitted filename: PONE-D-21-106788_Corrected.docx [file pone.0265716.s002.docx]

PLOS ONE

To: Plos One Journal Subject: letter for reviewer(s) or Submission of Manuscript

--Manuscript Draft--

| Manuscript Number: | PONE-D-21-10678 |
| --- | --- |
| Article Type: | Research Article |
| Full Title: | To: Plos One Journal Subject: letter for reviewer(s) or Submission of Manuscript |
| Short Title: | Magnitude And Factors Associated With Appropriate Complementary Feeding Practice |
| Corresponding Author: | kebede sadeta kumsa, MPH  Madda walabu university  Shashemene, west Arsi(oromia) ETHIOPIA |
| Keywords: | Appropriate complementary feeding practices, Children, Shashemene, Ethiopia |
| Abstract: | Abstract  Background  Inappropriate complementary feeding is a major contributor of child malnutrition. Over one third of under-five mortality is caused by malnutrition related to inappropriate complementary feeding. Thus this study designed to assess the prevalence and factors associated with appropriate complementary feeding practice among mothers of  children 6–23 months in Shashemene Town, Oromia Ethiopia.  Methods  Community based cross sectional survey was done from Feb-Mar. 2020.Study subjects comprised 536 mothers with 6-23months children selected using two stage sampling procedure. Data was analyzed using Statistical Package for the Social Sciences (SPSS) software. Logistic regression was used to identify factors associated with appropriate complementary feeding practice and statistical significance determined at the Pv of < 0.05.  Results  The proportion of 6-23months children who met the criteria for introduction of complementary food, minimum meal frequency, dietary diversity, acceptable diet and appropriate complementary feeding practices were 67.9 %, 61.7%,42.5%,41.7%, and  30% respectively.  The independent factors associated with appropriate complementary feed practice were child age 12-17and18-23 months [AOR: 2.32,95% CI: (1.40–3.82) ** 1.91(1.103.32) **]. socioeconomic status: mothers in wealth index of house hold, second, Middle, and fourth, [AOR: 4.27,95%, CI (1.8-10.22) ** 4.02(2.23-9.94) ** 7.02(3.2715.1) **], N o of ANC ≥4visit [AOR:2.57,95%CI:(1.3-5.05)] **, sources of information [AOR:3.5,95%, CI:(1.458.26) **], knowledge and positive attitude were found to be independent predictors of appropriate complementary feeding practice s among mothers of 6-23months children in Shashemene town [AOR: 2.34,95%, CI:(1.43-3.84 ]**, [ AOR:1.80,95%, CI:(1.15-2.82] * respectively.  Conclusion  This study demonstrated appropriate complementary feeding practice of children aged  6–23 months was low. Mothers who have children age 6–11 months, No of ANC ≥4visit, socioeconomic status, sources of information, mothers’ knowledge and positive attitude were significantly associated factors for appropriate feeding practices. Thus, nutritional education/counseling intervention on child feeding practices were recommended |
| Order of Authors: | kebede sadeta kumsa, MPH |
|  | Junayde Abdurahiman Ahmed |
|  | Kelil kenaoli Hussein |
| Additional Information: |  |
| Question | Response |
| Financial Disclosure  Enter a financial disclosure statement that | The funders had no role in study design, data collection and analysis, decision to publish, or preparation of the manuscript. |

| describes the sources of funding for the work included in this submission. Review the [submission guidelines](http://journals.plos.org/plosone/s/submission-guidelines#loc-financial-disclosure-statement) for detailed requirements. View published research articles from [PLOS ONE](http://journals.plos.org/plosone/) for specific examples.  This statement is required for submission and will appear in the published article if the submission is accepted. Please make sure it is accurate. |  |
| --- | --- |
| Unfunded studies Enter: The author(s) received no specific funding for this work. Funded studies Enter a statement with the following details: - Initials of the authors who received each award - Grant numbers awarded to each author - The full name of each funder - URL of each funder website - Did the sponsors or funders play any role in the study design, data collection and analysis, decision to publish, or preparation of the manuscript? - NO - Include this sentence at the end of your statement: The funders had no role in study design, data collection and analysis, decision to publish, or preparation of the manuscript. - YES - Specify the role(s) played. |  |
| * typeset |  |
| Competing Interests  Use the instructions below to enter a competing interest statement for this submission. On behalf of all authors, disclose any [competing interests](http://journals.plos.org/plosone/s/competing-interests) that could be perceived to bias this work—acknowledging all financial support and any other relevant financial or nonfinancial competing interests.  This statement is required for submission and will appear in the published article if the submission is accepted. Please make sure it is accurate and that any funding | he authors have declared that no competing interests exist. |

| sources listed in your Funding Information later in the submission form are also declared in your Financial Disclosure statement.  View published research articles from [PLOS ONE](http://journals.plos.org/plosone/) for specific examples. |  |
| --- | --- |
| NO authors have competing interests Enter: The authors have declared that no competing interests exist. Authors with competing interests Enter competing interest details beginning with this statement: I have read the journal's policy and the authors of this manuscript have the following competing interests: [insert competing interests here] |  |
| * typeset |  |
| Ethics Statement  Enter an ethics statement for this submission. This statement is required if the study involved:   - Human participants - Human specimens or tissue - Vertebrate animals or cephalopods - Vertebrate embryos or tissues - Field research   Write "N/A" if the submission does not require an ethics statement.  General guidance is provided below. Consult the [submission guidelines](http://journals.plos.org/plosone/s/submission-guidelines#loc-guidelines-for-specific-study-types) for detailed instructions. Make sure that all information entered here is included in the Methods section of the manuscript. | Ethical approval was taken from madda walabu university |

Format for specific study types

Human Subject Research (involving human

participants and/or tissue)

Give the name of the institutional review

board or ethics committee that approved the

study

•

Include the approval number and/or a

statement indicating approval of this

research

•

Indicate the form of consent obtained

(

written/oral) or the reason that consent was

not obtained (e.g. the data were analyzed

anonymously)

•

Animal Research (involving vertebrate

animals, embryos or tissues)

Provide the name of the Institutional Animal

Care and Use Committee (IACUC) or other

relevant ethics board that reviewed the

study protocol, and indicate whether they

approved this research or granted a formal

waiver of ethical approval

•

Include an approval number if one was

obtained

•

If the study involved

non-human primates

,

add

additional details

about animal welfare

and steps taken to ameliorate suffering

•

If anesthesia, euthanasia, or any kind of

animal sacrifice is part of the study, include

briefly which substances and/or methods

were applied

•

Field Research

Include the following details if this study

involves the collection of plant, animal, or

other materials from a natural setting:

Field permit number

•

Name of the institution or relevant body that

granted permission

•

Data Availability

Authors are required to make all data

underlying the findings described fully

available, without restriction, and from the

time of publication. PLOS allows rare

exceptions to address legal and ethical

concerns. See the

[PLOS Data Polic](http://journals.plos.org/plosone/s/data-availability)

[y](http://journals.plos.org/plosone/s/data-availability)

and

[FA](http://journals.plos.org/plosone/s/data-availability#loc-faqs-for-data-policy)

[Q](http://journals.plos.org/plosone/s/data-availability#loc-faqs-for-data-policy)

for detailed information.

Yes - all data are fully available without restriction

| A Data Availability Statement describing where the data can be found is required at submission. Your answers to this question constitute the Data Availability Statement and will be published in the article, if accepted. |  |
| --- | --- |
| Important: Stating ‘data available on request from the author’ is not sufficient. If your data are only available upon request, select ‘No’ for the first question and explain your exceptional situation in the text box. |  |
| Do the authors confirm that all data underlying the findings described in their manuscript are fully available without restriction? |  |
| Describe where the data may be found in full sentences. If you are copying our sample text, replace any instances of XXX with the appropriate details.   - If the data are held or will be held in a public repository, include URLs, accession numbers or DOIs. If this information will only be available after acceptance, indicate this by ticking the box below. For example: All XXX files   are available from the XXX database (accession number(s) XXX, XXX.).   - If the data are all contained within the manuscript and/or Supporting Information files, enter the following: All relevant data are within the manuscript and its Supporting Information files. - If neither of these applies but you are able to provide details of access elsewhere, with or without limitations, please do so. For example:   Data cannot be shared publicly because of [XXX]. Data are available from the XXX Institutional Data Access / Ethics Committee (contact via XXX) for researchers who meet the criteria for access to confidential data.  The data underlying the results presented in the study are available from (include the name of the third party | All relevant data are within the manuscript and its Supporting Information files. |

| and contact information or URL).  • This text is appropriate if the data are owned by a third party and authors do not have permission to share the data.  * typeset |  |
| --- | --- |
| Additional data availability information: | Tick here if your circumstances are not covered by the questions above and you need the journal’s help to make your data available. |

Manuscript	[Click here to access/download;Manuscript;manuscript.pdf](https://www.editorialmanager.com/pone/download.aspx?id=28412243&guid=f15ffaf9-48e7-41b3-a5f9-f61a1a8d2260&scheme=1)

Magnitude And Factors Associated With Appropriate Complementary Feeding Practice Among Mothers of Children 6–23 Months Age in Shashemene Town, Oromia- Ethiopia: Community Based Cross Sectional Study**.**

Junayde Abdurahmen^1^, Kebede Kumsa ^1^ and Kelil Hussein^2^

1. School of Public Health Shashamene campus of Madda Walabu

University,Oromia,Ethiopia. 2. School of Nursing Shashamene campus of Madda Walabu University, Oromia, Ethiopia.

* Corresponding authors: juneabdu@gmail.com (JA)

# Abstract

## Background

Inappropriate complementary feeding is a major contributor of child malnutrition. Over one third of under-five mortality is caused by malnutrition related to inappropriate complementary feeding. Thus this study designed to assess the prevalence and factors associated with appropriate complementary feeding practice among mothers of children 6–23 months in Shashemene Town, Oromia Ethiopia.

## Methods

Community based cross sectional survey was done from Feb-Mar. 2020.Study subjects comprised 536 mothers with 6-23months children selected using two stage sampling procedure.

Data was analyzed using Statistical Package for the Social Sciences (SPSS) software. Logistic regression was used to identify factors associated with appropriate complementary feeding practice and statistical significance determined at the Pv of ≤ 0.05.

## Results

The proportion of 6-23months children who met the criteria for introduction of complementary food, minimum meal frequency, dietary diversity, acceptable diet and appropriate complementary feeding practices were 67.9 %, 61.7%,42.5%,41.7%, and 30% respectively.

The independent factors associated with appropriate complementary feed practice were child age 12-17and18-23 months [AOR: 2.32,95% CI: (1.40–3.82) ** 1.91(1.10-3.32) **]. socioeconomic status: mothers in wealth index of house hold, second, Middle, and fourth, [AOR: 4.27,95%, CI (1.8-10.22) ** 4.02(2.23-9.94) ** 7.02(3.27-15.1) **], No of ANC ≥4visit [AOR:2.57,95%CI:(1.3-5.05)] **, sources of information [AOR:3.5,95%, CI:(1.458.26) **], knowledge and positive attitude were found to be independent predictors of appropriate complementary feeding practice**s** among mothers of 6-23months children in Shashemene town [AOR: 2.34,95%, CI:(1.43-3.84 ]**, **[**AOR:1.80,95%, CI:(1.15-2.82]***** respectively.

## Conclusion

This study demonstrated appropriate complementary feeding practice of children aged 6–23 months was low. Mothers who have children age 6–11 months, No of ANC ≥4visit, socioeconomic status, sources of information, mothers‟ knowledge and positive attitude were significantly associated factors for appropriate feeding practices. Thus, nutritional education/counseling intervention on child feeding practices were recommended.

### **Introduction**

#### Background

Children are most vulnerable to malnutrition in developing countries due to low dietary intakes, lack of appropriate care, and inequitable distribution of food within the household [1,2]. Appropriate feeding practices are important in improving nutritional status and child survival. Worldwide, two-thirds of the 10.9 million deaths annually among children under five are often associated with inappropriate feeding practices [3,4].

Complementary feeding refers to gradual dietary transition by introducing solid and semisolid foods to an infant‟s diet when breast milk alone becomes insufficient in meeting the nutritional needs of infant. The age range for complementary feeding is taken to be 6 to 24 months even though breastfeeding may continue beyond 2 years [3]. The world health organization (WHO) developed 8 core and 7 optional indicators to monitor and guide infant and young child feeding practices [5 ,6]. Ethiopia is one of the sub Saharan African countries with high level of malnutrition and has launched the national strategy for infant and young children feeding in 2004 to improve the nutritional status of children [7].

WHO recommends a combinations of indicators to measure level of appropriate complementary feeding, but, most studies conducted so far on complementary practices had used single indicator with narrow age range and not adequately quantify the level and determinants of appropriate complementary feeding practices [5,6].

Study conducted in Arsi nagele showed the level of appropriate complementary feeding was 9.5% [8]. Infant feeding practices are changing with time and place beside this there is a constraint of information on appropriate complementary feeding in developing countries which holds true for the study setting to explain the level of appropriate complementary feeding and associated factors. However, it has never been assessed in Shashemene town. Thus, the study designed to assess the prevalence of appropriate complementary feeding practices and associated factors among children aged 6–23monts. The findings would help to promote appropriate complementary feeding and it serves as base line information for any concerned bodies.

## General Objective

The objective of this study was to assess the prevalence of appropriate complementary feeding practices among mothers having 6-23 months children, from Feb.-march 2020 and associated factors in XX .

### Specific Objectives

To assess the prevalence of appropriate complementary feeding practice among mothers having 6-23 months children in XX

To identify factors associated with appropriate complementary feeding practices among mothers having children 6-23 months

### Operational Definitions

**Timely introduction of complementary feeding**: The proportion of children 6–23 months who were introduced to solid and semisolid foods at 6 months of age (5,6).

**Minimum dietary diversity:** is the proportion of children 6–23 months of age who receive foods from 4 or more food groups with the food groups consisting; (I) grains, roots and tubers; (II) legumes and nuts; (III) dairy products; (IV) flesh foods; (V) eggs; (VI) vitamin A rich fruits and vegetables; and (vii) other fruits and vegetables during the previous day of study [5,6].

**Minimum meal frequency:** is the proportion of breastfed and non-breastfed children 6–23 months of age, who receive solid, semi-solid, or soft foods (but also including milk feeds for non-breastfed children) the minimum number of times or more during the previous day(xx). **Minimum** is defined as 2 times for breastfed infants 6–8 months, 3 times for breastfed children 9–23 months, 4 times for non-breastfed children 6–23 months [5,6].

**Minimum acceptable diet:** is the proportion of children 6–23 months of age who receive both minimum meal frequency and minimum dietary diversity during the previous day of study. **Appropriate complementary feeding practice**: - defined appropriate when they meet all the four indicators timely introduction, minimum meal frequency and minimum dietary diversity and MAD while it is considered inappropriate when it fails to fulfill even a single indicator [5,6].

**Knowledge and attitude on ACFP** was measured among respondents using

- 10 items [(1). ever breast feed your child? (2). Time of initiation of first breast milk? (3) How long you gave Breast Milk for the baby? (4) having information on CF; (5). have knowledge about the advantage of iron rich food? (6). know the benefit of Iodized salt? (7). frequency of feeding solid, semisolid or soft food] (8) sources information about advantage of breast feeding? (9). sources information about commercially prepared complementary food? and (10). how to prepare complementary foods? and
- 4items [1. It is important to help my child when she eat, 2. It is important to feed my child to eat slowly and patiently,3. It is important to encourage my child to eat and 4.I talk to my child during feeding by looking straight in the eyes] respectively. For each item, , mothers responded
- „„Yes‟‟ were given a value of „„1,‟‟ while those cited „„No‟‟ were given „„0‟‟ and summed up. Those mothers scored mean or more were considered as having good knowledge, whereas those scored below mean value were characterized as poor knowledge similarly procedure were followed for attitude those who scored mean or more value were considered as having good attitude, whereas those scored below mean value were categorized as poor attitude towards ACFP. Conceptual frame work of complimentary feeding practice from different literature review ,2020(**Figure** 1).

## Methods

### Study settings and period

Shashemene is the most densely populated town in oromia region, with diversified ethnic groups of population. it is located at 250 km from Finfinne, the capital city of Ethiopia. Climatically, Shashemene town falls into subtropical climatic zones: The Population of Shashemene town is estimated to be 272193: 50.4% male and 49.6% are female in 2019 Children aged 6–23 months of age constituted 4.8 % of population i.e.13065 as to Shashemene health office report of 2020. The study was conducted in Shashemene Town, from Feb. – march 2020**.**

### Study design and population

A community based Cross sectional study was conducted in Shashemene town Oromia, Ethiopia from Feb-March.in 2020. All Mothers who had children aged 6–23 months residing in

Shashemene town by 2020 were considered as source population whereas mothers of children aged 6–23 months living in selected households during the study period and resided in the study area for more than 6 months was taken as the study population. Mothers who resided in the study area for <6 months were excluded from the study subjects.

Inclusion and exclusion criteria

All mothers-child pairs age 6-23 months living in Shashamene town was taken as source population. Whereas mothers – child pairs aged 6–23 months living in selected household during the study period as well resided in the study area for ≥6 months presented during the study period were included as study population while

### Independent and dependent variables

The outcome variable for this study was the magnitude of ACFP and its components. The independent variables were maternal, child and household characteristics. Briefly, the description of the variables was as follows: Sociodemographic characteristics: -age, sex, family size, monthly income, Partner education level, household wealth index, food insecurity, occupation, residence, knowledge, and attitude of mothers, Obstetric history:( Pregnancy History, ANC, PNC, delivery mode and Place of delivery, birth space and # of Parity)

### Sample Size determination

Sample size were determined by single population proportion formula considering the proportion of appropriate complementary feeding practice 11.4% from previous study [9]. were sampled.

 »» n=

The following assumptions were used:

- Margin of error=4%;
- Zα =1.96 and
- Design effect =2.

243 samples were obtained with consideration of 10% contingency to non-responders a total of 536 mothers were sampled.

### Sampling procedure

A two stage sampling technique was used to select the study subjects. Four (4) sub citiesy was randomly selected using simple random sampling method from 8 sub cities. The total population in the four (4) selected sub cities: Bulchana, Arada, Alelu and Awasho was 139510 (respectively 36877, 34529 ,31734 and 36370 of which 6696 was children of 6–23 months of age. The calculated sample (536) was allocated equally among the selected 4 sub cities i.e. 134 mothers having children 6– 23 months in each sub city. To get the individual sample units or subjects at household level all target group of the sub city was obtained from the health post before calculating K then determined K.

The random start was identified by lottery method and every Kth mother with eligible children was picked from 4 sub city using systematic random sampling thus a child was selected in each sub city and his/her mother was interviewed accordingly. From each household one eligible child with mother at the time of survey was selected, incase ≥2 children found the younger was taken and the process had been continuing until next Kth in the same direction. If a mother was absent on the date of data collection she was substituted by the next mother from the same sub city after one revisit.

### Data collection instruments

Data were collected by using face-to- face interview during home to home visit to mothers who had children aged 6–23 months using structured questionnaire. The questionnaire comprised of information on background characteristics of mother and children, maternal health practice, and child feeding practices.

### Data collection Methods

Data was collected by using face-to- face interview during home-to-home visit from mothers who had children aged 6–23 months using structured questionnaire. The questionnaire comprised of information on background characteristics of mother and children, maternal health practice, and child feeding practices. Six diploma holder data collectors and two BSc. holder supervisors were recruited. For data quality control, the questionnaire was first developed in English and translated to local language, Afan Oromo, and then back translated to English by two people, who have good command in both languages for consistency. Training was given to data collectors and supervisors for 2 days and the questionnaire was pre-tested in 5% of mothers, in the study area, which is not included in actual study to assess the content and approach of the questionnaire and necessary correction was made. All questionnaires were checked on daily bases for completeness while data was thoroughly checked and cleaned before analysis.

### Data processing and analysis

Wealth index was computed as a measure of household wealth using principal component analysis (PCA). Fifteen variables related to ownership of selected household assets, size of quantity of durable equipment‟s, materials used for housing construction, and ownership, improved water and sanitation facilities were considered. Finally, the generated principal component was divided into 5 equal quintiles (lowest, second, middle, fourth, and highest).

Data was coded, entered and analyzed using statistical package for social science (SPSS) version 25. Descriptive statistics such as frequencies, proportions, means, and standard deviation are performed to describe data. Bivariate analysis was made to appreciate the relation of each independent variable with the dependent variable. Finally, independent variables associated during bivariate analysis with P-value ≤0.25 was entered in a multivariable logistic regression analysis used to determine the strength of association between independent and dependent variables. Odds ratios along with 95% CI was reported and the statistical significance was declared at the p-value <

0.05. Multivariate Logistic regression model was used to control confounders.

### Ethical considerations

The research proposal was reviewed and approved by the research and ethics committee of the MWU. Permission was obtained from relevant authorities. An informed consent form was made available to the study participants. The consent was in line with the ethical principle of

“autonomy” that gives participants the right to stop participating in the study. Finally, information regarding importance of appropriate CF practice and nutritional advice will be given to respondents by interviewers. Furthermore, intervention strategies will be designed to take appropriate action

Result of the study	Socio demographic Characteristics of Study Subjects***.***

In this study from 536 sampled mothers, 520 mother-child pairs were participated in the study giving the response rate of 97.01%. The mean age of mothers was 26.83 ±4.41 years. More than half of study subjects were between the age range of 25–34 years. Regarding educational status, about 22% of mothers had basic education only where as 14.8% of the households has no work. Of the total study subjects, 481 (92.5%) were married, Live at different place and not married each 14(2.7%), Divorced10(1.9%) and widowed 1(.2%). 325 (62.5%) Muslim by religion and More than half 333(64%) of the respondents were Oromo in ethnic group (**Table**-**1).**

Table 1: Socio demographic characteristic of mothers with children 6-23 month, Shashemene	town, Ethiopia, 2020. (n=520)	ETB=Ethiopian birr

| **Age of mothers/caretakers** | **No** | **percent** |
| --- | --- | --- |
| < =24 years | 151 | 29.4 |
| 25–29 years | 231 | 44.4 |
| 30–34 years | 100 | 19.2 |
| 35+ | 38 | 7.3 |
| **Religion of mothers** |  |  |
| Orthodox | 95 | 18.5 |
| Protestant | 81 | 15.6 |
| Catholic | 13 | 2.5 |

| Others | 6 | 1.2 |
| --- | --- | --- |
| **Educational status of Mothers** |  |  |
| Basic Education | 117 | 22.5 |
| Primary school | 236 | 45.4 |
| Secondary school | 113 | 21.7 |
| Higher Education | 54 | 10.4 |
| **Educational status of Fathers** |  |  |
| Basic Education | 71 | 13.7 |
| Primary school | 189 | 36.3 |
| Secondary school | 192 | 36.9 |
| Higher Education | 68 | 13.1 |
| **Mothers/caretakers occupation** |  |  |
| Housewife | 244 | 46.9 |
| Daily Labor | 150 | 28.8 |
| Gov‟t employee | 64 | 12.3 |
| Students | 18 | 3.5 |
| Merchant | 44 | 8.5 |
| **Husband’s occupation** |  |  |
| Gov‟t employee | 149 | 28.7 |
| Farmer | 77 | 14.8 |
| Merchant | 217 | 41.7 |
| Daily laborer | 28 | 5.4 |
| Others | 49 | 9.4 |
| **Family income per month** |  |  |
| < =999 ETB | 13 | 2.5 |
| 1000–1999 ETB | 84 | 16.2 |
| 2000–2999 ETB | 129 | 24.8 |
| 3000–3999 ETB | 89 | 17.1 |
| > =4000 ETB | 205 | 39.4 |

| **Ethnicity** | | |  |
| --- | --- | --- | --- |
| Oromo | 333 | | 64 |
| Amhara | 89 | | 17.2 |
| Wolaita | 47 | | 9 |
| Others | 51 | | 9.8 |
| **Family size** | | |  |
| 1–3 | 101 | | 19.4 |
| 4–6 | 327 | | 62.9 |
| > =7 | 92 | | 17.7 |
| **Who decide on the properties of the Household** | | |  |
| Husband | 166 | | 31.9 |
| Wife | 31 | | 6 |
| Jointly | 323 | | 62.1 |
| **Sources of information about commercially available CF** | | |  |
| HCWs | 95 | | 18.3 |
| Family | 163 | | 31.3 |
| Media | 215 | | 41.3 |
| Relative | 16 | | 6.9 |
| Others | 11 | | 2.1 |
| **Sources of information about Advantage of Breast Milk** | | |  |
| TV | 77 | | 14.8 |
| Radio | 97 | | 18.7 |
| Health professional | 237 | | 45.6 |
| Reading | 54 | | 10.4 |
| Family/relative | 55 | | 10.6 |
| **Knowledge about advantage of iodized salt** | | |  |
| Yes | 365 | | 70.2 |
| No | 155 | | 28.8 |
| **Knowledge about advantage of Iron ric**h **food** | |  |  |
| Yes |  | 267 | 51.3 |
| No |  | 253 | 48.7 |
| **Source of drinking water** | |  |  |
| Piped into dwelling/Public tap |  | 505 | 97.1 |
| Bottled water |  | 15 | 2.9 |
| **Household wealth index** | |  |  |
| Lowest |  | 13 | 2.5 |
| Second |  | 61 | 11.7 |
| Middle |  | 203 | 39 |
| Fourth |  | 151 | 29 |
| Highest |  | 92 | 17.7 |
| **House Hold food security** | |  |  |
| Secured food |  | 407 | 78.3 |
| In secured |  | 113 | 21.7 |

Abbreviation ???

Most of study participants 351(67.5%) were Multiparous and 494(95%) of them attended antenatal care. Four hundred fifty (86.5%) of the respondents‟ birth interval from previous birth were greater than 2years (**Table -2).**

Table 2: Maternal obstetric related characteristics of mothers of children 6-23 month Shashamene town, Oromia, Ethiopia, 2020.

| **Parity** | **No** | **Percent** |  |
| --- | --- | --- | --- |
| Prim parous (1) | 92 |  | 17.7 |
| Multiparous (2–4) | 351 |  | 67.5 |
| Grand multipara (5+) | 77 |  | 14.8 |
| **Delivery attendants** |  |  |  |
| HCW | 460 |  | 88.5 |
| TBAs‟ | 35 |  | 6.7 |
| Other‟s | 25 |  | 4.8 |
| **Mode of delivery** |  |  |  |

| Normal | 468 | 90 |
| --- | --- | --- |
| C/S | 49 | 9.4 |
| Others | 3 | 0.6 |
| **Place of delivery** |  |  |
| Home | 94 | 18.1 |
| Health Facilities | 426 | 81.9 |
| **No ANC attendants** |  |  |
| No ANC session | 26 | 5 |
| <=3 | 279 | 53.7 |
| >=4 | 215 | 41.3 |
| **Attend ANC services** |  |  |
| Yes | 494 | 95 |
| No | 26 | 5 |
| **Attend PNC** |  |  |
| Yes | 288 | 55.4 |
| No | 232 | 44.6 |
| **Time of PNC attendance** |  |  |
| Not attend | 231 | 44.4 |
| 0-2days | 128 | 24.6 |
| 3-6days | 127 | 24.4 |
| 7days & above | 34 | 6.5 |
| **Birth Spacing** |  |  |
| <2 | 338 | 65 |
| 2-4 | 160 | 30.8 |
| >4 | 22 | 4.2 |
| **No of Children in the family** |  |  |

| 1-3 | 384 | 73.8 |
| --- | --- | --- |
| 4-6 | 120 | 23.1 |
| >6 | 16 | 3.1 |
| **Birth order** |  |  |
| 1st | 101 | 19.4 |
| 2nd | 141 | 27.1 |
| 3rd | 152 | 29.2 |
| 4th | 59 | 11.3 |
| 5th &above | 67 | 12.9 |
| **Sex of the child** | |  |
| Male | 262 | 50.4 |
| Female | 258 | 49.6 |
| **Age of the child** | |  |
| 6-8Months | 93 | 17.9 |
| 9-11Months | 70 | 13.5 |
| 12-17Months | 209 | 40.2 |
| 18-23Months | 148 | 28.5 |
| **Did the child have diarrhea in the last 2Week?** | |  |
| Yes | 239 | 46 |
| No | 281 | 54 |
| **Did the Child had ARTI** | |  |
| Yes | 292 | 56.2 |
| No | 228 | 43.8 |
| **Did the Child had Fever** |  |  |
| Yes | 329 | 63.3 |
| No | 191 | 36.7 |

Almost all mother 519(99.8%) BF their children after delivery indicating that very young children are mostly fed breastmilk, as recommended. 496/520(95.5%) initiated BF earlier. i.e. 272 (52.4%) give breast milk immediately after birth, 224(43.1%) within one hour, the rest 23(4.4%) started within 24 hr. and most of them 482(92.7%) feed breast milk based on child demand.

Thirty-one (6%) mothers start CF earlier. The most common reasons for early initiation of CF were mothers‟ negative attitude towards the quantity of breast milk, not stay with the child and lack of knowledge about breast milk. The median duration and the mean duration of any BF in Ethiopia are 25 months. fig.3 below, indicates mothers BF status, the median duration of BF was 14months.i. e 243/520(46.7%) lower than the EDHS,2011 (**fig.3).**

### Food and fluid provided for the children

The most commonly provided food or fluids for the children were milk of any types 417(80.2%) followed by potatoes 324(62.3%), porridge/gruel 235(45.2%) and Bread, vegetables and fruits 181 (34.8%).xx The mostly preferred food for the child was home made that accounts for 398 (76.5%) while the rest 122(23.5%) was preferred commercially available food.

Regarding CF practices and frequency during illness,187(36%) mothers decrease the quantity and the frequency of food,147(28.3%) withheld quantity and frequency of food ,123(23.7%) maintain same quantity and frequency while only 63(12. %) mothers used to increase food quantity and frequency. Despite cultural and social food restriction commonly on Cabbage and Meat which accounts for 124(23. 8%).Concerning food preparation out 520 mothers,452 (86.9%) prepare separately for child while 68(13.1%) prepared with adult food.

Grains, roots and tubers, dairy products and Vitamin A rich foods were the most commonly taken food items among all age categories children in previous 24hr preceding the survey. Legumes and nuts, Eggs and Vitamin A rich fruit and vegetables were consumed higher among age 18-23 months of children‟s compared to the other groups in 24 hr preceding survey. Eggs were the least consumed in age group 6-11months however, there was less consumption of flesh food across all age categories in contrary to other food groups (**Table**- **3**).

Table 3: Types of food groups given during the previous day according to age of the child among children aged 6–23 months, Shashemene, 2020 (n= 520) Vit = vitamin A

| Food groups | Age of children in months | | | | | | | | | | | |
| --- | --- | --- | --- | --- | --- | --- | --- | --- | --- | --- | --- | --- |
|  | 6–11 month (163) | | | | 12–17 month (209) | | | | 18–23 month (148) | | | |
|  | Yes | (%) | No | (%) | Yes | (%) | No | (%) | Yes | (%) | No | (%) |
| Grains, roots & tubers | 135 | **82.8** | 28 | 17.2 | 187 | **89.5** | 22 | 10.5 | 131 | **88.5** | 17 | 11.5 |
| Legumes and nuts | 65 | **39.5** | 98 | 60.1 | 94 | **45** | 115 | 55 | 84 | **56.8** | 64 | 43.2 |
| Dairy products | 107 | **65.6** | 56 | 34.4 | 139 | **66.5** | 70 | 33.5 | 91 | **61.5** | 57 | 38.5 |
| Egg | 71 | **43.6** | 92 | 56.4 | 110 | **52.6** | 99 | 47.4 | 90 | **60.8** | 58 | 39.2 |
| Flesh foods | 20 | **12.3** | 143 | 87.7 | 39 | **18.7** | 170 | 81.3 | 39 | **26.4** | 109 | 73.6 |
| Vit. A rich foods | 90 | **55.2** | 73 | 44.8 | 116 | **55.6** | 93 | 44.5 | 108 | **73** | 40 | 27 |
| Other fruits and vegetable | 77 | **47.2** | 86 | 52.8 | 75 | **35.9** | 134 | 64.1 | 73 | **49.3** | 75 | 50.7 |

### Complementary feeding status‟ Indicators

CF was assessed on five hundred twenty mothers-child pairs enrolled in the study. Out of this three hundred fifty-three (67.9%) of the mothers had introduced for CF to children at 6 months‟ as suggested while 31(6%) before 6 months and the rest late after 6 months. Introduction of solid, semi-solid or soft foods by age 6-8months was, (92.5%) but vary across age from 112/209 (53.6%)of 12-17m, 53/70 (75.7%) of 9-11m, 12-15m-(68.9%), 1823m (68.9%). Over all, three hundred twenty-one 321(61.7%), mothers fed their children meeting

MMF as recommended. Overall, less than half (41.7%) of children aged between 6 - 23 months met the requirement for MAD was (31.2%.) in the age group of 6- 9 months. The highest proportion of mothers who fed their children MAD was in the age group 1823months which was 78(52.7%). . however,. The level of ACFP by combining the four indicators was 156(30%)

#### (Figure 4)

Slightly more than half of the children aged 9–23 months that was 112/190 (53·1 %), 256/399, (64.2%), and 260/399 (65.2%). met ACFP, MDD, and MAD criterion for BF Children respectively. Eighty three 83 /93 (89.2 %) Mothers fed their children one or two times among breastfed child 6-8 months‟ age whereas 313(73.3%) for breast-fed child 9-23months age fed three or more times. The proportion of BF mothers with child age of 6-23months who fed their children and met the MMF, MDD, and MAD criteria were 376/489(76.9%),261/489, (53.4%), and 327/489, (66.9%) respectively. Similarly, the proportion of BF mothers with 9 to 23 months who met MMF was 307/399(76.9%). Nearly more than ¾, of children aged 6– 8 months (76.7%) had met MMF for BF children. Of 31 Non BF children of age 6-23months only 21/31(67.7%),14/31(45.2%),10/31(32.3%) met the criteria for MMF, MDD and MAD respectively.

**Knowledge and Attitude toward ACFP**

Overall, 319 (61.3%) of the respondents had good knowledge about ACFPWith regard to attitude about ¾ of participants 391 (75.2%) had positive attitude toward ACFP,

#### Bivariate and Multivariable Analyses

Table 4 illustrated factors associated with ACFP among mothers having children aged 6–23 months were identified: A Variables having P.value less than 0.25 in bivariate analyses were maternal education, occupation, parity, birth interval, family size, household income, child age, place of delivery, maternal ANC, No of ANC, PNC, sources of information, wealth index, food security issues, knowledge and attitude. In this study no association was found between maternal occupation and education, birth interval, family size, ANC, place of delivery, PNC and ACFP.

Of the total entered variables, the finding revealed socio economic status (wealth index, household food security, household income), sources of information, child age, No of ANC follow up, knowledge and attitude are factors significantly associated with ACFP while the rest variables were not associated after controlling for potential confounders though associated in bivariate analyses.

Only antenatal care follow-up of four times and above was found to be statistically significantly associated with appropriate complementary feeding practice mothers whose

ANC No ≥ 4 times were 2.6 times more likely to practice appropriate complementary feeding than those counterpart (AOR = 2.57, 95% CI: 1.3, 5.05).

Household socio economic status (wealth index and food security) was found to be statistically significantly associated with complementary feeding practices. mothers who were in the second, middle, fourth and highest percentile xx were about four times more likely to practice appropriate complementary feeding than those who were in lowest percentile, (AOR = 4.27, 95% CI: (1.8-10.22) **, 4.02,95% CI: (2.23-9.94) **and 7.0, 2,95% CI: (3.27-15.10) ****** respectively. After controlling for potential confounders, our results reveal that mothers who belonged to a food secure household were 1.7 times more lokely to practice appropriate complementary feeding than the mothers in food insecure househo.ld [(AOR = 1.71,95%, CI: (1.07-2.75 **)].

Children whose mothers get information regarding breast feeding from health workers were 3.5 times higher tobenefite from ACFP compared with children whose mothers got from other sources of information (AOR = 3.5,95 %, CI: 1.45-8.26) **).

Mothers with children in age categories (12–17m) and18-23 months showing an association in practicing ACFP compared with the other groups children [AOR=2.32,95%, CI:(1.40–3.82) **.and AOR, =95, CI:1.91(1.10-3.32)] **. Result also revealed that knowledge and attitude about ACFP of the mothers was significantly associated with ACFP. Children from households with mother having positive attitude were 1.8 times more likely to practice ACFP compared to those from mothers with negative attitude (AOR = 1.80, 95% CI = (1.15, 2.82)) *. Similarly, the odd of practicing ACFP among mothers how had good knowledge was 2.34 times higher than that of the counterpart [AOR=2.34,95 %, CI:(1.43-3.84). ** (**Table -4**).

Table 4: Factors associated with complementary feeding of children age 6–23 months, Shashamene Oromia, Ethiopia, 2020 .

| Variables | Complementary feeding | | COR (95 % CI) | AOR (95 % CI) |
| --- | --- | --- | --- | --- |
|  | Appropriate | Inappropriate |  |  |
| Maternal education | No (%) | No (%) |  |  |
| No formal education | 39(25) | 78(21.4) | 1 | 1 |
| Primary school | 73(46.8) | 163(44.8) | 0.90 (.57–1.43) | .92(.57-1.48) |
| ≥2^ry^ school | 63(34.2) | 104 (31) | 1.08 (.67-1.77) | 1.27 (.076-2.13) |

| Maternal occupation Housewife | 87(55.8) | 157 (43.1) | 1 | 1.00 |
| --- | --- | --- | --- | --- |
| Daily Labor | 32(20.5) | 118 (32.4) | 1.26(.82-1.92) | 0.82(0.37-1.81) |
| Gov‟t | 16(10.3) | 48(13.2) | 1.50(.82- 2.63) | 1.94(.82-4.57) |
| Students | 9(5.8) | 9(2.5) | .59(.19--1.84) | 1.58(.60-4.22) |
| Merchants | 12(7.7) | 32(8.8) | 1.17(.60-2.29) |  |
| Sex of the child |  |  |  |  |
| Male | 72(46) | 190(52.2) | 1.04(.73-1.50) | .77(1.05-74) |
| Female | 84(53.8) | 174(47.8) | 1 | 1 |
| Family size  1–3 | 40(21.7) | 61 (18.2) | 2.37 (0.89–6.33) | 0.78 (.29–2.06) |
| 4–6 | 104 (56.5) | 223 (66.4) | 2.68 (1.17–6.13) | 0.88 (0.38–2.44) |
| ≥7 | 40(21.7) | 52(15.5) | 1.00 | 1.00 |
| Family income/monthly  ≤999 | 6(3.8) | 7 (1.9) | 6.32 (0.78-51.10) | 6.58 (.79–54.53) |
| 1000–1999 ETB | 22(14.1) | 62 (17) | 5.59(.70–44.46) | 7.19(0.88–58.72) |
| 2000–2999 ETB | 44 (28.2) | 85(23.4) | 8.15(1.02–65.47)* | 10.10 (1.21–84.19)* |
| 3000–3999 ETB | 27(17.3) | 62(17) | 7.22 (0.92–56.60) | 10.02(1.23–81.26)* |
| 4000+ ETB | 57(36.5) | 148 (40) | 1.00 | 1.00 |
| Age of the child  6–8 months | 40(21.7) | 53 (15.8) | 1 | 1.00 |
| 9–11 months | 25 (38.6) | 45 (13.4) | 1.39 (.82-2.37) | 1.40 (0.77–2.56) |
| 12-17months  18–23 months | 67(36.4)  52(28.3) | 142 (42.3)  96(28.6) | 2.75 (1.2–11.80) *  . 1.81(1.15- 2.85) ** | 2.32, (1.40–3.82) **  1.91(1.10-3.32).** |
| Place of delivery  Health facility | 118 (75.6) | 308(84.6) | .83(.52-1.33) | 1.18(.39-2.47) |
| Home | 38 (24.4) | 56 (15.4) | 1.00 | 1.00 |
| No of ANC attendance |  |  |  |  |
| No ANC | 7(4.5) | 19(5.2) | 1 | 1 |
| ≤3 | 62(39.7) | 217(59.6) | 1.22(0.61-2.40) | 1.14(0.57-2.29) |
| >=4 | 87(55.8) | 128(35.2) | 2.72(1.40-5.29)** | 2.57(1.3-5.05)** |
| Attended ANC  Yes | 56 (98.2) | 494 (89.2) | 6.80 (0.92–50.03) | 0.82(.33-2.03) |
| No | 1 (1.8) | 60 (10.8) | 1.00 | 1.00 |
| Attended PNC  Yes | 86 (55.1) | 202(55.5) | .96 (.67-1.38) | 1.04 (0.69–1.358) |
| No | 70(44.9) | 162(44.5) | 1.00 | 1.00 |
| Parity of the mother Prim parous (1) | 21(13.5) | 71(19.5) | 2.00 (0.72–5.55) | 2.38(.75-7.53) |
| Multipara (2–4) | 115(73.7) | 236(64.8) | 2.89 (1.27–6.59) | .87(.35-2.18) |
| Grand multipara (5+) | 20 (12.8) | 57(15.7) | 1.00 | 1.00 |
| No of children |  |  |  |  |
| 1-3 | 125(80.1) | 259(71.2) | 1 | 1 |
| 4-6 | 25(16) | 95(26.1) | 81(.29-2.29) | 1.34(.34-5.13) |
| >6 | 6(3.8) | 10(2.7) | 1.28(.44-3.73) | 1.63(.50-5.27) |
| Birth interval |  |  |  |  |
| ≤2 | 103(66) | 235(64.6) | 1 | 1 |
| 3-4 | 45(28.8) | 115(31.6) | 1.57(1.07-2.32) | .97(.36-2.63) |
| >4 | 8(5.1) | 14(3.8) | 1.22(.50-2.99) | 1.52(.57-4.06) |
| House hold Wealth index |  |  |  |  |
| Lowest | 1(0.5) | 12(3.6) | .68(.08-5.83) | .63(.07-5.56) |
| Second | 22(12) | 39(11.6) | 4.63(2.0-10.71)** | 4.27(1.8-10.22)** |
| Middle | 80(43.5) | 123(26.6) | 5.33(2.61-0.90)** | 4.02(2.23-9.94)** |
| Fourth | 71(38.6) | 80(23.8) | 7.28(3.51-5.11)** | 7.02(3.27-15.1)** |
| Highest | 10(5.4) | 82(24.4) | 1 | 1 |
| House Hold Food Security |  |  |  |  |
| Secured | 111(71.2) | 296(81.3) | 1.59(1.01-2.52)** | 1.71(1.07-2.75)** |
| Not Secured | 45(28.8) | 68(18.7) | 1 | 1 |
| Sources of information |  |  |  |  |
| TV | 17(10.9) | 60(16.5) | 1 | 1 |
| Radio | 36(23.1) | 61(16.8) | 2.08(1.06-4.10)* | 1.67(.77-3.65) |
| HCWs | 27(17.3) | 27(7.4) | 3.53(1.65-7.53)** | 3.5(1.45-8.26)** |
| Reading | 55(35.3) | 182(50) | 1.07(.58-1.98) | 1.02(.49-2.08) |
| Relative/family | 21(13.5) | 34(9.3) | 2.18(1.01-4.68)* | 2.36(.96-5.77) |
| Mothers attitude |  |  |  |  |
| Positive | 108(69.2) | 283(77.7) | 1.55(1.02-2.36)* | 1.80(1.15-2.82* |
| Negative | 48(30.8) | 81(22.3) | 1 | 1 |
| Mothers‟ Knowledge |  |  |  |  |
| good Knowledge | 107(68.6) | 212(58.2) | 1.57(1.05-2.33)* | 2.34(1.43-3.84)** |
| poor knowledge | 49(31.4) | 152(41.8) | 1 | 1 |

Were are the abbreviations related to the better understanding of this table

### Discussion

The percent study was conducted on magnitude and factors associated with appropriate complementary feeding practice (ACFP) which had a response rate of 97.01%. Over all, the result revealed that the magnitude of ACFP was 30%, this is higher than that of study done in Arsi Negele,9.5%, Damota sore 11.4%, and Ghana 14.3% [ 8-10] but lower than that of Sri Lankan 68%, Bangladeshi 40% and Nepal 32% [11,12]. This is possibly due to difference in study setting, socio economic status or indicators used to measure appropriate complementary feeding or sociocultural differences among different population at different time.

In the recent study, the lowest proportion of children who had been fed with ACFP was in the age group of 6-11months compared with the counter group this imply the need of giving due attention for the younger age. In this study the proportion of children who had been introduced to solid, semi-solid or soft foods among all aged 6–23 months was 67.9 % while that of 6-8 months was 86/93, (92.5%). This is higher than that of Arsi nagele,72.5%, Damota sore 74.2%, Ghana 72.6%, Nepal and Bangladesh studies reported with 70–71 % [8-12]. This figure is coinciding with WHO recommendation of more than 80 % of 6–8 months‟ children should initiate complementary feeding at 6 months of age [5,6]. Nevertheless, in the current finding, the time of introduction of complementary feeding is better than other similar studies conducted elsewhere [8,10.13,14]. Maternal Health care services accessed such as ANC, PNC and institutional delivery services utilization and extensive effort of HEWs, health development army beside information differences in the study area were better in the present study that could be resulted in better awareness and practices on appropriate time of complementary feeding introduction compared with other studies. Other explanations could be the difference in time dimension and study setting socio- cultural diversity.

The proportion of children aged 6–23 months who met the MMF criteria was (61.7 %). It is nearly comparable with finding from Arsi Nagle (67.3 %) and in Bale Zone Ethiopia (68.4%)

[8,15]. However, it‟s lower than studies conducted in Sri Lanka (88.3 %), Bangladesh (81 %), Nepal (82 %), coastal South India (77.5 %) Derashe, Southern Ethiopia (95 %) and Amibara district, North East Ethiopia **(**69. 2%) **[**11,12,17-19]. This disparity might be due to sociocultural, educational and working conditions of care givers.

With regard to minimum dietary diversity(MDD) the current study also revealed that the proportion of children who met MDD was 42.5%, this reflects as only this mothers fed their young children with four or more food groups from seven food sets i.e. grains, roots and tubers; legumes and nuts; dairy products; flesh foods; vitamin A reached food; eggs, and other fruits vegetable which is almost similar with that of Bangladesh 42%, but higher than the fig. stated from studies done in, Arsi Nagle

18.8 %, Damota sore 16%., India 15% ,Nepal 34%, however it‟s lower than that of Sir Lanka 71%

[8,9,11,12,20]. While in Eastern and South Africa it was one in ten infant and young children [21].

The high variation from Damota sore and Arsi nagle could be due to the current study was done in the city where there was better in information and maternal health care access led to difference in awareness of the mothers and educational status variations whereas the previous studies were done in rural areas where mothers were less advantageous compared to the urban counterpart. The low consumption of protein-rich foods can be due multifold factors including lack of nutritional awareness and shortage of access due to economic constraints [22].

The percent study revealed the varieties of foods given to younger children is lower and MDD only tends to increase with growing age (table3), i.e. the lowest proportion who met MDD was found in the age group of 6-11months (fig.4). Similar patterns have also been observed in Ethiopia and other developing countries [8, 22-24]. This might be because mothers may assume that younger infants do not need diversified food or their gut may not be able to digest animal source foods besides this the most commonly restricted food is meat and cabbage hence mothers believe that children can‟t be swallowed. Moreover, flesh food is the least consumed food across all age while eggs were the least consumed in age group 6-11months. Subsequently, CF might be initiated with monotonous staples. This is in line with a study from Northern

Ethiopia which found that flesh foods and eggs were introduced in children‟s diets in the middle of the second year of age [16]. The study observed that household economic status as measured by wealth index and food security level was a significant predictor of MDD. Obviously, the lower economic status restricts the availability and variety of food in the household.

Minimum acceptable diet incorporates MMF and MDD, was 41.7%. This is comparable with finding from Bangladesh 40%, Ghana 46% where MAD [10-12]. But this is higher than that of Ethiopia national level 7%, and Abiy Adi, North Ethiopia 11.9%, India 9%, Nepal 32%, But, it was lower than the finding from Sirlanka 68% [25, 11,12,16]. The lower level of the result might be due to socio economic -cultural, policy differences among the study area and time. Thus, the low prevalence of this indicator proposes that majority of children were either not fed as frequently as the recommended 2-4 times daily, or were not offer food from four or more the recommended food groups in their diet, this may result in inappropriate CF practices which led to malnutrition. High MAD in this study was observed as compared to the National figure 7 % as of EDHS 2016.

This might be because of EDHs was a nationally representative survey with a wide range of child feeding styles in different parts of the regions of Ethiopia with diversified sociocultural context beside this the DHS covers nationwide both rural and urban areas that reduce the figure. The higher figure observed in our study may be due to current expansion of HEWs in the study area that focused on ANC, PNC and child care education which in turn increases maternal exposure to healthcare workers so that increases their practices. Moreover, this is pocket study that localized in to town where there is better access and availability to information, education, health care services utilization and other social services across time variation.

Over all the proportion of children who met ACFP was 30%. This was higher than that of the result from Arsi Negele 9.5%, Damota sore,11.4%, Abiy Adi town, North Ethiopia where ACFP was 10.5% [8,9, 16]. This might be due to differences in study setting hence the previous study was done in rural parts of the country where there is low access to maternal health care services and media.

**Factors associated with Appropriate Complementary Feeding Practices**

The current study revealed that household socio economic status (wealth index and food security status, household income), sources of information, No of ANC follow up, child age good knowledge and positive attitude are factors significantly associated with ACFP while the rest variables were not associated or lost association after controlling for potential confounders though associated in bivariate analyses.

Only ANC follow-up of four times and above was found to be statistically significantly associated with ACFP i.e. those mothers whose ANC No ≥ 4 times were 2.6 times more likely to practice ACFP than those counterpart (AOR = 2.57, 95% CI: 1.3, 5.05**). This result is similar with the findings in Damota sore, Sirlanka, Nepal, and India where fewer antenatal clinic visits were associated with delay in the introduction of complementary food [9-12] ANC contacts ≥ 4time were a significant predictor of ACFP. This might be due to the effects of information and counseling that the mothers received from health care providers during their antenatal care services utilization. This is evidenced by those mothers who get in contact with health care workers were significantly associated with ACFP (AOR=3.5,95%, CI:(1.45-8.26) **. This may indicate that promoting the utilization of maternity services and stronger integration with IYCF helps to improve infant feeding practices.

One more important determinant factor associated with ACFP was household economic status as measured by wealth index and food security level. Household wealth index was found to be statistically significantly associated with CF practices. Those mothers who‟s in highest percentile: the second, middle, fourth were about four times more likely to practice ACFP than those who were in lowest percentile (AOR = 4.27, 95%, CI: (1.8-10.22) **, 4.02,95% CI: (2.239.94) **and 7.0, 2,95% CI: (3.27-15.10) ****** respectively. So, this illustrates economically higher peoples are more likely to practice ACFP than the counter parts. in fact, higher household wealth index positively associated with a higher dietary diversity score (DDS). Families under the high percentile are more likely to be able to afford and offer a variety of foods to their children more frequently. The positive association between household with highest wealth percentile and an increased diet diversity has been consistently reported in previous studies done in Sri Lanka, Pakistan, Indian, Bangladesh and Nepal [11,12]. Which was consistent with our finding.

The global nutrition report 2018 also revealed diets of infant and young children are suboptimal everywhere in all wealth groups from 75.6% in lowest to 56.7% highest quintile [26]. The fact that household wealth is a predictor of MDD underlines the important role of household assets in determining optimal CF practices [11,12] which in line with our finding. This study disagreed with the findings from Arsi Nagele, Damota sore Ethiopia, Nepal and Sirlanka where maternal education is the predictors of ACFP [8,911,12,16,]. The possible explanations can be the difference in societal norms and cultures with geographic difference regarding female education.

Child age is also found to be predictor variables as older children (12–23 months) are about three times more likely to be feed appropriately compared with younger children (6–11 months). Similarly, studies conducted in five Asian countries and Tanzania, Arsi nagele and Northern part of Ethiopia reported child age as a predictor variable [8, 11,12,16, 20,]. This might give an opportunity for the health planners to give due attention for younger children feeding.

Global nutrition report indicated 74.6% of children 6–23 months of age do not have sufficient diet diversity for a healthy diet worldwide hence inappropriate CF after 6 months of age is one major cause of malnutrition, in turn malnutrition is the leading cause of the global burden of disease so attention should be given to young child nutrition education intervention to meet

SDG Agnda „refers to ending “all forms of malnutrition‟‟ [26].

Other important predictor variable was attitude. Positive Mother‟s attitudes show statistically significant with regarding ACFP. This may be the effect of counseling the mothers received from health care providers during their antenatal care services utilization. The most important issues on feeding during illness was only 63(12. %) mothers were increase food quantity and frequency. Despite cultural and social food restriction commonly on Cabbage and meat which need due attention. One more important variable was mothers‟ knowledge where those mother who had good knowledge regarding ACFP shows significant difference against those who had poor knowledge this in line with study done Tehran [27]. This might be due to the information provided during ANC attendant that accounts 95% in this study.

Interestingly, there are encouraging practices detected in this study: - almost all mother 519(99.8%) breast feed their children after delivery, 496/520(95.5%) initiated Breast Feed earlier, and most of them 482(92.7%) feed breast milk based on child demand, whereas 489(94%) fed EBF vs 58% national fig. of 2016 EDHS. It is recommended that a child continues to breastfeed until age of 2year. However, in Ethiopia, the percentage of children who are currently breastfeeding decreases from 91 % among children age 12-17 months to 76 % among children age 18-23 months as of EDHS 2016 that is a bit comparable with this finding.

The most commonly preferred food for the child was homemade among 398 (76.5%) while the rest 122(23.5%)did not. In contrary, the discouraging practices were thirty-one (6%) mothers start CF earlier, breast fed continuation was not as recommended at 1&2 years / I.e. not satisfactory but by far better than that of Nigeria and Kosovo [28,29]. The most common reasons for early initiation of CF were mothers‟ negative attitude towards the quantity of breast milk, not stay with the child and lack of knowledge about breast milk. Nearly 488 (86.2%) mothers used bottle feeding, whereas during illness only 63(12 %) mothers were increase food quantity and frequency while the rest 88%did not. Despite cultural and social food restriction commonly on Cabbage and Meat which accounts for 124(23. 8%). 452 (86.9%) prepare food separately while 68(13.1%) prepared with adult food. Overall, Low level of ACFP, MDD, MAD was observed in this study particularly among children <11months age**, So**, it need mitigation to improve CF. Limitations

Provided that it was cross-sectional: collects point in time data as well it doesn‟t show cause effect relationship. The study was done in the town it may not give the full picture of the areas rather this may inflate the results. Furthermore, the figure might have also been over- or underestimated due to recall and social desirability bias that may introduced to the time of initiation, diversity and frequency of food. The 24 h dietary diversity recall may show only the current feeding and needs repeated measures. Other drawback of our study may be generalizability, hence our study was done among the population from a single town that may not be representative of the region.

### Conclusion and Recommendation

To this end, the overall prevalence of ACFP was low which have an impact on the health of infants and young children. This‟s show the importance of immediate action to promote ACFP. The proportion of mothers met MMF, MDD, and MAD was also low despite, high timely introduction of complementary feeding. Child age, ≥4 ANC follow up, household socioeconomic status, sources of information, mothers‟ knowledge and positive attitude were factors significantly associated with ACFP.

### Recommendation

It was stated in previous studies as 2/3 of child death was attributed to inappropriate complementary feeding, in this study ACFP was low. So, to scale up these successful interventions to levels that would make an impact. Health Bureau, NGOs and other development sectors should give special attention to:

Educational/ counselling intervention on nutrition for mothers and /or caregivers are essential for improving infant and young child feeding practices, particularly for mothers with younger children about, time, variety, quantity and frequency of food.

Promoting the socioeconomic status of the community particularly for poor mothers with households with lowest wealth index /food insecure via multiple discipline/inter-sectoral collaboration engagement to improve ACFP.

HCWs should encourage the women to attend more ANC as well as give attention to mothers during counselling on Infant nutrition /educational interventions to improve complementary feeding practices and reduce malnutrition.

To come up with the real figure large scale longitudinal study will be proposed for the researcher

### Supporting information

Figure 1: Conceptual frame work of complimentary feeding practice from different literature review, 2020.

Figure 2: Diagrammatic presentation of sampling scheme of the sampled mothers-child pairs of 623months age Shashamene, Ethiopia, 2020.

Figure 3: Indicator of breast feeding status among 6-23 month age children in shashemene oromia, Ethiopia, 2020.

### Acknowledgements

The investigators would like to thanks Madda Walabu University, Shashemene Campus for offering the fund to carry out this research project that is targeted to find out community problems. Materials used in this project are honorable acknowledged. The investigators express their sincere appreciations to peoples and organization (Shashemene Health Bureau, Shashemene City Administration for offering necessary information that help to conduct the research.

Lastly, we would like to present our heartfelt thanks to data collectors and study participant for support they provided us throughout the whole process of data collection period.

### **Author Contributions**

Junayidi Abdurahaman and Kebede Kumsa were involved in proposal writing, designed the study and participated in coordination, supervision and the overall implementation of the project, analyzed the data and finalized the Manuscript. Kelil Hussein conceived the study and participated in all stages of the study and revision of the manuscript.

All authors read and approved the final manuscript

#### 8. References

1. Yalew BM. Prevalence of malnutrition and associated factors among children age 6-59 Months at Lalibela town, north Wollo Zone, northern Ethiopia. J. Nutr Disorders ,2014.
2. Semahegn A, Tesfaye G, Bogale A. Complementary feeding practice of mothers and associated factors in Hiwot Fana specialized hospital, eastern Ethiopia. Pan Afr Med J. 2014; 818:143
3. Guiding principles for complementary feeding of the breastfed child. Division of health promotion and protection. Pan American health organization/WHO. Washington/Geneva: PAHO, WHO; 2003.
4. Mancharia CW, Kogi-Makau W, Muroki NM. Dietary intake, feeding and care practices at children in kathonzweni, division, Makuenl, district, Kenya. East Afr Med J.,2004
5. WHO. Indicators for assessing IYCFP. Part 1: definitions. Geneva: WHO; 2008
6. WHO, UNICEF, USAID, FANTA, AED, UC DAVIS, and IFPRI. Indicators for assessing I YCFP part 2: measurement. Geneva: The WHO; 2010.
7. Disha A, Rawat R, Subandoro A, Menon P. IYCF practices in Ethiopia and Zambia and their association with child nutrition: analysis of demographic and health survey data. Afr J Food Agric Nutr Dev.,2012
8. Tigist Kassa, Berhan Meshesha, Yusuf Haji and Jemal Ebrahim (2016). Appropriate complementary feeding practices and associated factors among mothers of children age 6–23 months in southern Ethiopia, 2015 Pediatrics,16:131 DOI 10.1186
9. Abate	Areja, Dereje	Yohannes	and	Mulugeta	Yohannis.	Determinants	of	appropriate	complementary feeding practice among mothers having children 6–23 months of age in rural Damot sore district, Southern Ethiopia; BMC Nutrition (2017) 3:82 DOI 10.1186
10. Abukari I, Issaka, Kingsley E Agho, Penelope Burns, Andrew Page^1^ and Michael J Dibley. Determinants of inadequate complementary feeding practices among children aged 6–23 months in Ghana, University of Sydney, Sydney, New South Wales, Australia. doi:10.1017/S1368980014000834,2014
11. Senarath	U,	Agho K, Akram D,	Godakandage	S,	Hazir	T,	Jayawickrama	H,	et	al. Comparisons of complementary feeding indicators and associated factors in children aged 6–23 months across five south Asian countries. Maternel Child Nutr. 2012, Jan;8 Suppl 1:89-106
12. Senarath U & Michael J. Dibley_._ Complementary feeding practices in South Asia: analyses of recent national survey data by the South, Asia Infant Feeding Research Network. Maternl& Child Nutr. Blackwell Publishing Ltd (2012), **8** (Suppl. 1), pp. 5–10
13. Tessema M, Belachew T, Ersino G. Feeding patterns and stunting during early childhood in rural communities of Sidama, South Ethiopia. Pan Afr Med J. 2013; 14
14. Melkam A, Molla M, Zelalem B, Azeb A. Dietary Diversity and Meal Frequency Practices among Infant and Young Children Aged 6–23 Months in Ethiopia: 2 ^ry^ Analysis of EDHS 2011. J Nutr Metab. 2013; Vol. (2013):8.
15. M. Tegegne, S. Sileshi, T. Benti, M. Teshome, and H. Woldie, “Factors associated with minimal meal frequency and dietary diversity practices among infants and young children in the predominantly agrarian society of Bale zone, Southeast Ethiopia: a community based cross sectional study,” *Archives of PH*, vol. 75, no. 1, pp. 1–11, 2017
16. Ergib M, Shumey A, Ferede S, Haile F. Magnitude and factors associated with appropriate complementary feeding among mothers having children 6–23 months-of-age in Northern Ethiopia; A Community-Based Cross-Sectional Study. J Food Nutr Sci. 2014;2(2):36.
17. Rao S, Swathi PM, Unnikrishan B, Hegre A. Study of complementary feeding practices among mothers of child aged six months to two years- A study from coastal south India. Australasian Med J. 2011;4(5):252–7,
18. Wondafrash M, Amsalu T, Woldie M. Feeding styles of caregivers of children 6-23 months of age in Derashe special district, Southern Ethiopia. BMC Public Health. 2012; 12:235
19. Mohammed Wagris, Anwar Seid, Molla Kahssay and Osman Ahmedk8. Minimum Meal Frequency Practice and Its Associated Factors among Children Aged 6–23 Months in Amibara District,North East Ethiopia
20. Victor R, Baines SK, Agho KE, Dibley MJ. Factors associated with inappropriate complementary feeding practices among children aged 6-23 months in Tanzania. Matern Child Nutr. 2012;10(4):545–61
21. UNICEF. Infant and Young Child Feeding; Current Status	progress. 2016
22. S. Gebremedhin, K. Baye, T. Bekele et al., “Level and predictors of dietary diversity Children 6– 23 months of age in predominately food insecure area of South Wollo, Ethiopia,” *Nutr.*, vol. 33, pp. 163–168, 2017.
23. D. Dangura and S. Gebremedhin, “Differentials of dietary diversity in children 6– 23 months in Gorche district, southern Ethiopia,” *BMC Pediatrics*, vol. 17, p. 9, 2017
24. V. Khanal, K. Sauer, and Y. Zhoa, “Determinants of complementary feeding practices among Nepalese children aged 6–23 months: findings from demographic and health survey 2011,” *BMC Pediatrics*, vol. 13, no. 1-131, 2013
25. EDHS, 2015. Addis Ababa and Calverton: CSA and ORC Macro; 2016
26. Corinna Hawkes. Center for food policy, City university of London. Global nutrition report 2018, 27. Shams N, Mostafavi F, Hassanzadeh A. Determinants of complementary feeding practices among mothers of 6 – 24 months’ failure to thrive children based on behavioral analysis phase of precede model,Tehran. 2016.Int. J. of Homeopathy & Natural Medicines ,2019; 5(1): 1-7 7
28. Okwori E, Onu R, Onagwa GI, et al. Infant feeding practices and the effect of early complementary feeding on child nutritional status in Makada, Sabon Gari Local Gov’t Area, Kaduna State, Nigeria. Nigerian J. of Nutritional Sciences 2011:32
29. Merita Berisha, Naser Ramadani, Rina Hoxha, Sanije Gashi, Valbona Zhjeqi, Drita Zajmi, Ilir

Begolli: Knowledge, Attitudes and Practices of Mothers in Kosova About Complementary

Feeding for Infant and Children 6-24 Months. 2017

Supporting Information

[Click here to access/download](https://www.editorialmanager.com/pone/download.aspx?id=28412537&guid=4787a0cd-54c9-4d11-b226-37d19501a89e&scheme=1)

[Supporting Information Acronum.pdf](https://www.editorialmanager.com/pone/download.aspx?id=28412537&guid=4787a0cd-54c9-4d11-b226-37d19501a89e&scheme=1)
