# Supplementary material for: Magnitude and factors associated with appropriate complementary feeding practice among mothers of children 6–23 months age in Shashemene town, Oromia- Ethiopia: Community based cross sectional study
Source: PLoS One. 2022 Mar 29;17(3):e0265716. doi: 10.1371/journal.pone.0265716 (PMC8963544; doi:10.1371/journal.pone.0265716)
Supplement: Attachment — Submitted filename: Response to submission PONE-D-21-10678R2.docx [file pone.0265716.s004.docx]

01-3-2022

To: PLOSONE journal

Subjects: Authors Response to Reviewer(s) “**Manuscript N^o^ “PONE-D-21-10678R1”**

Dear/Sir,

I sincerely hope that you are doing well. Thanks very much for your very valuable and constructive comments that you support to our manuscript. We have deeply appreciate for your remarkable contribution in providing valuable comments and offering you precious time that you invests to look at this manuscript “We Thanks so much more again for your nobility and patience”. While we are looking at your comments we found that the comments were very caring for improving our manuscript. Thus, we addressed as per the provided comments/suggestions by the reviewer(s) stated below as follow:

With regard to the legend, under each or within the tables or figures abbreviations were stated briefly for the reader to avoid confusion. You may look at each fig. and tables. Thanks for your nice feedback. Other comments were also addressed accordingly, it was marked with green color in manuscript with track change, and so, would you mind looking at the change?

Table 1: legends indicated **in line N^o^ 283 would see it please**: ETB =Ethiopian Birr, TV: Television, CF: complementary feeding, HCWs: health care workers

Table 2: The legends indicated **in line N^o^ 293-295.**it was marked with green color in the manuscript with track change. So, would see it pleas: TBAs=Traditional Birth Attendants, TBAs=Traditional Birth Attendants, HCW=health care workers, ANC (Antenatal care), C/S=Cesarean Section.

**Table 3: similar you may see line 332-333, Vit = vitamin A**

**Table 4:** **line N^o^ 416-419** *=stands for value of ≤0.05, **=stands for P. vale of less than or equal to 0.001,TV: television ,HCWs: health care workers, ANC: Antenatal care, PNC: Postnatal care, ETB: Ethiopian birr, COR:, crud odd ratio, AOR: Adjusted Odd ratio, CI: confidence interval

**Figure 1:** CFP: Complementary feeding practices, #: number, ANC: Antenatal care, PNC: Postnatal care

**Figure 2: legends were written in line 214-215.** D/Boke: Didaboke, pop(n): Population, B/gudina: Burka Gudina, m: month

**Figure 3:** legends indicated in line N^o^ **305-306, would you see it was indicated in manuscript with track change as** BF: Breast feeding, IYCF: Infant and young child feeding, BM: breast milk, EBF: exclusive breast feeding

**Figure 4:** legends indicated in line N^o^ **305-306, would you see,** ACFP: Appropriate complementary feeding practice. Again it was added under the supporting information **line N^o^. 633-634.would you refer it please?**

**Best regards**

Junyde Abdurahmen
